# Resolvin E1 improves efferocytosis and rescues severe aplastic anemia in mice

**DOI:** 10.1038/s41419-024-06705-7

**Published:** 2024-05-09

**Authors:** Rachel Grazda, Allison N. Seyfried, Krishna Rao Maddipati, Gabrielle Fredman, Katherine C. MacNamara

**Affiliations:** 1 Department of Immunology and Microbiology, Albany Medical College, Albany, NY USA; 2 Department of Pathology, Lipidomics Core Facility, Wayne State University, Detroit, MI USA; 3 Department of Molecular and Cellular Physiology, Albany Medical College, Albany, NY USA; 4 Present Address: Institute for Clinical Pharmacodynamics, Schenectady, NY USA

**Keywords:** Chronic inflammation, Mechanisms of disease

## Abstract

Severe aplastic anemia (SAA) is a rare, fatal disease characterized by severe cytopenias and loss of hematopoietic stem cells (HSCs). Immune-mediated destruction and inflammation are known drivers of SAA, however, the underlying mechanisms driving persistent inflammation are unknown. Current treatments for SAA rely on immunosuppressive therapies or HSC transplantation, however, these treatments are not always effective. Using an established mouse model of SAA, we observed a significant increase in apoptotic cells within the bone marrow (BM) and impaired efferocytosis in SAA mice, relative to radiation controls. Single-cell transcriptomic analysis revealed heterogeneity among BM monocytes and unique populations emerged during SAA characterized by increased inflammatory signatures and significantly increased expression of *Sirpa* and *Cd47*. CD47, a “don’t eat me” signal, was increased on both live and apoptotic BM cells, concurrent with markedly increased expression of signal regulatory protein alpha (SIRPα) on monocytes. Functionally, SIRPα blockade improved cell clearance and reduced accumulation of CD47-positive apoptotic cells. Lipidomic analysis revealed a reduction in the precursors of specialized pro-resolving lipid mediators (SPMs) and increased prostaglandins in the BM during SAA, indicative of impaired inflammation resolution. Specifically, 18-HEPE, a precursor of E-series resolvins, was significantly reduced in SAA-induced mice relative to radiation controls. Treatment of SAA mice with Resolvin E1 (RvE1) improved efferocytic function, BM cellularity, platelet output, and survival. Our data suggest that impaired efferocytosis and inflammation resolution contributes to SAA progression and demonstrate that SPMs, such as RvE1, offer new and/or complementary treatments for SAA that do not rely on immune suppression.

## Introduction

Idiopathic severe aplastic anemia (SAA) is a rare form of bone marrow failure (BMF) characterized by hematopoietic stem cell (HSC) loss and pancytopenia [1, 2]. Patients with SAA have significantly increased IFNγ and TNF in the blood and BM [3, 4]. While thought to be a disease driven by HSC destruction via auto-reactive T cells, additional cell types contribute to pathogenesis. Macrophages were found to be necessary for disease progression via IFNγR- and CCR5-dependent signals [5, 6], and macrophage-derived TNF was critical for T cell recruitment to the BM and production of IFNγ during SAA [2]. Current therapies for SAA remain limited to targeting T cells via immunosuppressive therapies (IST; anti-thymocyte globulin and cyclosporine) and BM transplantation [7–9], though new therapies are needed.

The frequency of apoptotic CD34^+^ progenitor cells is increased in the BM of SAA patients, and frequencies of apoptotic cells correlate with disease severity [10]. Rampant cell death, driven by Fas/FasL, leads to the destruction of HSCs and other cells [11]. Although macrophages are critical for promoting inflammation and SAA progression [2, 5, 6], macrophages are also important phagocytes within the BM where they remove apoptotic cells [12, 13] and promote tissue regeneration [14]. Efficient clearance of apoptotic/dead cells by phagocytes, termed efferocytosis, is crucial for inflammation resolution and tissue function at homeostasis [15]. Apoptotic cells provide “find-me” and “eat-me” signals that ensure swift engulfment [16]. However, cell clearance is tightly regulated to prevent aberrant removal of healthy cells, and “don’t eat-me” signals, such as CD47, interact with inhibitory receptors like SIRPα to inhibit efferocytosis [17, 18].

Inflammation resolution is an active process mediated specialized pro-resolving lipid mediators (SPMs). SPMs are derived from omega-3 polyunsaturated fatty acids and possess potent pro-resolving actions, including enhancing efferocytosis [19]. The SPM Resolvin E1 (RvE1) improved efferocytosis in various inflammatory disease models of both infectious and non-infectious origins [20–25]. Using an established murine model of SAA, we demonstrate dysfunctional efferocytosis and imbalanced pro-inflammatory and pro-resolving lipid mediators. Impaired efferocytosis correlated with a unique population of SIRPα^hi^ monocytes and an accumulation of CD47^+^ apoptotic cells in the BM. RvE1 treatment improved efferocytosis and provided significant protection against disease parameters, including death, in mice with established SAA. Our findings provide new insight to dysregulated inflammation resolution programs in SAA pathogenesis offering novel therapeutic strategies for BMF that improve inflammation resolution without limiting host defense.

## Methods

### Mice

Animal protocols were approved by Institutional Animals Care and Use Committee at Albany Medical College. C57BL/6 (H^b/b^) and BALB/cAnN (H-2^d/d^) mice were purchased from Taconic (Albany, NY). Macrophages insensitive to IFNγ (MIIG) mice were a gift from Dr. Jordan. Hybrid B6 F1 (H-2^b/d^) mice were generated by crossing C57BL/6 or MIIG mice (C57BL/6 background) with BALB/c mice.

### Bone marrow failure induction

Hybrid F1 mice were subjected to sub-lethal radiation (300 RADs,^137^Cs source) and received 6.5 × 10^7^ C57BL/6 splenocytes from age- and gender-matched donors via intraperitoneal injection [26, 27].

### Cell preparation and flow cytometry

Blood was collected from euthanized mice into EDTA-coated tubes via cardiac puncture and analyzed (Heska Element HT5). BM was flushed from femurs and tibias. After RBC lysis, cell suspensions were plated and stained (Supplementry Table 1). Data were collected using a FACSymphony A3 (BD Biosciences) with FACSDiva software or Cytek Northern Light (Cytek Biosciences) with SpectroFlow software and analyzed using FlowJo software (TreeStar, Ashland, OR). Imaging flow cytometry was performed using Amnis Imagestream-X Mark II (Cytek Biosciences) and analyzed using IDEAS software (EMD Millipore, Burlington, MA).

### Analysis of phagocytosis and efferocytosis

Anti-SIRPα (clone P84; 200μg; BioXCell) antibody was administered to mice via intraperitoneal injection on days 7 and 9 post induction for day 10 harvest, or days 7, 9, 11, and 13 for day 14 harvest. Fluorescent Dil (DilC_18_[3])-labeled liposomes (200 µL; Liposoma) were administered to mice via retro-orbital I.V. injection on day 9 post induction. BM and blood were harvested 16 h post injection. For efferocytosis assays, whole BM was flushed and incubated with 3 µM-sized phosphatidylserine (PS)-coated lipid microparticles (Echelon Biosciences) for 1 h. BM cells were incubated with staurosporine for 3 h and stained with CFSE or pHrodo^TM^ Red (Invitrogen). Labeled apoptotic cells were fed to whole BM and incubated for 3 h. Cells were treated with 1 nM RvE1, 5 μg/mL anti-CD47, or 10 μg/mL anti-SIRPα neutralizing antibody prior to incubation.

### Gene expression

Whole BM was flushed and pooled from hind limbs of three mice per group. RBCs were lysed and cells were stained to sort purified monocytes (CD11b^+^Ly6C^hi^Ly6G^-^), mRNA was isolated (Qiagen RNeasy Mini Kit), and quantitative-RT-PCR was performed (Eppendorf *realplex*^*2*^ Mastercycler). For single-cell analysis, whole BM was flushed and 7-AAD^-^CD11b^+^Ly6C^+^Ly6G^-^ cells were sorted on a BD FACSAria^TM^. Cells from each experimental group (radiation and SAA, *n* = 3 mice per group) were pooled. Sample preprocessing for sequencing was performed using Chromium Next GEM Single-Cell 5′ kit (10x Genomics). Sequencing and genome alignment were performed by the Center for Functional Genomics at SUNY Albany. The human BM dataset was obtained from Tonglin et al., GSE181989 [28]. An age-matched healthy and SAA patient was selected for analysis. Count matrices were loaded into R (version 4.3.1) using standard Seurat workflow. Cells with >25% mitochondrial RNA or <1000 detected genes were removed. Integration was performed utilizing the reference mapping approach described in Stuart et al. [29].

### RvE1 treatment

Resolvin E1 (250 ng; Cayman Chemical) was administered to mice via intraperitoneal injection every other day, starting day 7. For survival studies, mice were monitored twice daily for 28 or 37 days and euthanized when moribund. Euthanasia criteria was based on signs of dehydration, response to physical stimuli, and mobility — as previously described [6].

### Lipidomic analysis

Whole BM was flushed from femurs and tibias, flash-frozen on dry ice, and shipped to Wayne State University Lipidomics Core facility for analysis. Fatty acyl lipidomic analysis was performed as per published procedures [30, 31]. Samples were homogenized using Zirconium beads (Precellys, Biotage) and the homogenate was extracted for fatty acyl lipids using StrataX columns (Phenomenex) following supplementation with internal standards. Extracts were analyzed by LC–MS/MS using Multiple Reaction Monitoring method on a QTRAP5500 mass analyzer (Sciex). Identities of individual lipid mediators were confirmed from retention times and spectra recorded for each detected peak and were quantified relative to internal standards. Data were normalized against protein content of the samples (ng/mg protein).

### Statistical analysis

Data were analyzed with GraphPad Prism software (version 10.2.2, La Jolla, CA). Details on statistical analysis are provided in each figure legend. For lipidomics, principal component analysis was performed using 22 lipid mediators detected across all samples from mice 3 days post radiation for healthy, radiation controls, and SAA-induced mice. Data were standardized and principal components were selected using Kaiser–Guttman’s rule. Heat map analysis was performed on LC-MS/MS data from radiation controls and SAA-induced mice, normalized to healthy controls.

## Results

### Monocytosis, cytopenias, and increased marrow cell death during SAA

**Fig. 1:**
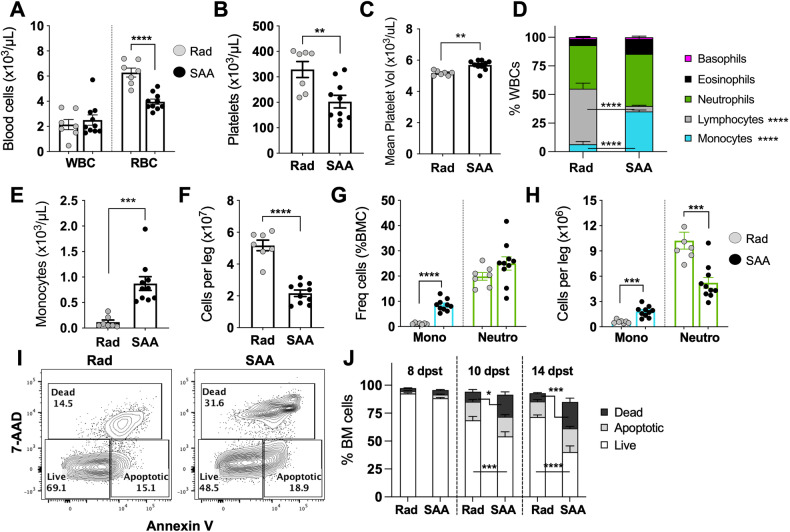
Cytopenias and BM hypocellularity are associated with increased cell death in SAA. F1 hybrid mice were induced to develop severe aplastic anemia via the radiation followed by splenocyte transfer model. Mice were euthanized 10 days post splenocyte transfer (dpst) and complete blood count data are shown for total WBCs and RBCs (**A**), platelets (**B**), and mean platelet volume (**C**). The breakdown of all WBCs (**D**) and total blood monocytes (**E**) is shown. **F** Overall BM cellularity in radiation control and SAA mice. **G** Frequency of BM monocytes (CD11b^+^Ly6C^hi^Ly6G^-^) and neutrophils (CD11b^+^Ly6C^lo^Ly6G^+^). **H** Absolute number of BM monocytes and neutrophils. Data shows two pooled independent experiments showing mean ± SD, *n* = 7–10 per group, significance was determined using a Student’s *t*-test. **p < 0.01, ***p < 0.001, ****p < 0.0001. **I** Gating strategy for Annexin V and 7-AAD staining to differentiate live, apoptotic, and dead BM cells, plots representative for radiation control and SAA mice. Numbers reflect the percent of cells within the gated region. **J** Frequency of BM cells that are live (open), apoptotic (gray), or dead (black) for both radiation controls and SAA mice days 8, 10, and 14 post splenocyte transfer. Data show two pooled independent experiments per time point showing mean ± SD, *n* = 5–10 per group, significance was determined using a Student’s t-test. *p < 0.05, ***p < 0.001, ****p < 0.0001.

Thrombocytopenia and BM hypocellularity are well-known characteristics of severe aplastic anemia (SAA) [1, 5, 6]. Using a murine model of SAA induced by adoptive transfer of splenocytes to sub-lethally irradiated F1 mice [5, 26, 32], we observed a significant decrease in red blood cells (Fig. 1A) and platelets (Fig. 1B) by 10 days post splenocyte transfer (dpst). Mean platelet volume was significantly higher in SAA mice as compared to radiation controls (RC) (Fig. 1C). Mice exhibited lymphopenia, however the proportion and absolute number of circulating monocytes was significantly increased in SAA (Fig. 1D, E). Frequencies and numbers of BM monocytes (CD11b^+^Ly6C^hi^Ly6G^-^) were also increased during SAA, despite extensive BM hypocellularity. In contrast, neutrophils (CD11b^+^Ly6C^lo^Ly6G^hi^) were decreased (Fig. 1F–H; Supplementry Fig. 1). Analysis of monocyte gene expression indicated an increase in *Ifng* and *Tnf*, known drivers of disease [1, 2], in SAA mice (Supplementary Fig. 2). Therefore, disease progression correlated with a hematopoietic program favoring monopoiesis.

SAA is characterized by BM aplasia, and to investigate kinetics of cell death during disease, we performed Annexin-V and 7-AAD staining (Fig. 1I). Similar frequencies of apoptotic (AnnV^+^7-AAD^−^) and dead (7-AAD^+^) cells were observed between RC and SAA-induced mice at 8 dpst (Fig. 1J). However, by 10 dpst SAA-induced mice had significantly increased dead cells and this was even more striking by 14 dpst. IFNγ can induce apoptosis [33] and transgenic mice containing macrophages insensitive to IFNγ (MIIG mice) exhibit protection from SAA [5]. SAA-induced MIIG mice exhibited a decrease in the frequency of dead cells in the BM, relative to littermate control (LC) (Supplementry Fig. 3), further demonstrating that accumulation of dead cells correlated with SAA progression.

### Single-cell transcriptomics reveals heterogeneity among BM monocytes

**Fig. 2:**
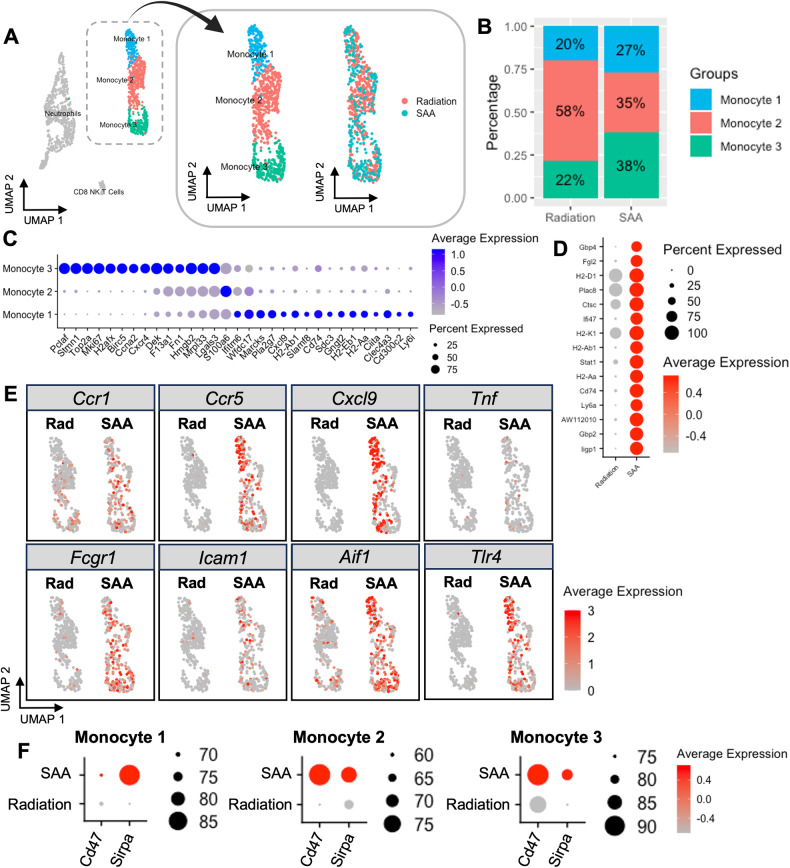
Single-cell sequencing reveals heterogeneity among BM monocytes. **A** UMAP plot of all cell clusters and extraction of three distinct monocyte clusters identified in BM samples from radiation control and SAA-induced mice. **B** Proportion and distribution of monocyte subsets in each sample. **C** Dot plot of the top differentially expressed genes in each monocyte population compared to the others. The size of the dot corresponds to the percentage of cells expressing each gene, while the color represents the average gene expression level. **D** Dot plot of top differentially expressed genes in SAA BM monocytes, compared to radiation control. **E** Feature plot of inflammatory gene expression in radiation control and SAA. **F** Dot plot of *Cd47* and *Sirpa* expression on each monocyte population.

The bias towards monocyte production and accumulation of dead cells suggested defects in BM monocyte lineage cells that impair clearance. Therefore, we next analyzed BM monocytes by performing single-cell RNA sequencing (scRNA-seq) on sorted BM monocytes. A total of 798 cells from RC and 825 cells from SAA mice were sequenced, and cells classified as monocytes were extracted from the dataset (Supplementry Fig. 4A–C, Fig. 2A). We observed three distinct monocyte clusters, of which, monocyte population 2 (Mono2) made up a majority (58%) in RC mice. The Mono2 population decreased significantly during SAA (35%), while both monocyte 1 (Mono1) and monocyte 3 (Mono3) increased (Fig. 2B). Utilizing differential gene analysis, we observed that Mono1 was enriched for antigen processing/presentation (*H2-Ab1, H2-Eb1, H2-Aa, Ciita)*, while Mono3 was enriched for proliferation/cell cycle genes (*Pclaf, Stmn1, Top2a, Ccna2*; Fig. 2C).

Consistent with the role of IFNγ as a main driver of disease, monocytes from SAA mice were strongly enriched for various interferon-stimulated genes (*Fgl2, Ifi47, Stat1, Ly6a, Gbp2, Gbp4, Iigp1)* (Fig. 2D). Many genes associated with inflammation, such as *Ccr1*, *Ccr5*, *Cxcl9*, *Tnf*, *Tlr4*, *Fcgr1*, *Icam1*, and *Aif1*, were highly upregulated in SAA (Fig. 2E, Supplementary Fig. 4D), suggesting that monocytes may perpetuate inflammation and contribute to disease progression.

Our observation that dead cells accumulated in the BM of SAA-induced mice suggested that efferocytosis may be impaired in SAA. Healthy cells actively suppress their engulfment via “don’t eat-me” markers, such as CD47, that bind inhibitory receptors on phagocytes, including SIRPα. As cells undergo apoptosis they lose or alter CD47 expression enabling phagocytic clearance [34, 35], and the CD47-SIRPα axis has been associated with impaired efferocytosis in several disease contexts [36–38]. Our scRNA-seq dataset revealed that all monocyte populations significantly upregulated *Cd47* and *Sirpa* during SAA (Fig. 2F). These data suggest that increased CD47 and SIRPα may contribute to the accumulation of dead cells.

To establish the clinical relevance of our murine model we analyzed a human BM scRNA-seq dataset from a healthy and SAA patient [28]. Consistent with our murine model, monocytes were increased in the SAA patient (Supplementry Fig. 5A). The extracted monocytes also exhibited an increase in inflammatory genes (*CCR1, TNF, ICAM1, AIF1, TLR4*, and, *CX3CR1*), and robust increase in *SIRPA* and *CD47* (Supplementry Fig. 5B–D). Together, these data support the role of increased monocytes and the CD47-SIRPα axis in the progression of SAA.

### Increased surface expression of SIRPα and CD47 during SAA

**Fig. 3:**
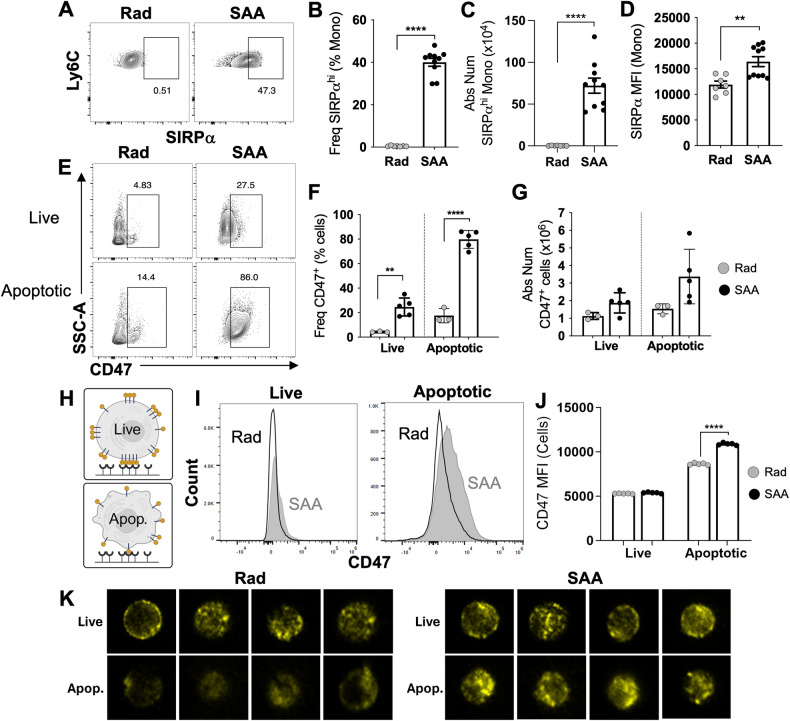
The expansion of SIRPα^hi^ monocytes and CD47 on apoptotic cells in SAA mice. Mice were induced to develop SAA and BM harvested 10 dpst. **A** Representative gating for SIRPα^hi^ monocytes. The frequency, absolute number, and MFI of SIRPα^hi^ monocytes are shown in **B**, **C**, and **D** respectively. Data represents two pooled independent experiments showing mean ± SD, *n* = 7–10 per group, significance using a Student’s *t*-test. **p < 0.01, ****p < 0.0001. **E** Gating strategy for CD47 on live and apoptotic BM cells. Frequency (**F**) and absolute number (**G**) of CD47 for live and apoptotic cells. Data representative of one experiment showing mean ± SD, *n* = 3–5 per group. Significance was determined using a Two-way ANOVA with Tukey’s multiple comparison test. **p < 0.01, ****p < 0.0001. **H** CD47 expression and clustering on live versus apoptotic cells. **I** Histogram of CD47 fluorescence intensity on live and apoptotic BM cells. **J** CD47 MFI on live and apoptotic BM cells. **K** Representative cells from imaging flow cytometry of live (AnnV^−^) and Apoptotic (AnnV^+^) BM cells stained with CD47 (yellow).

To validate our scRNA-seq analysis, we examined monocytes and macrophages by flow cytometry and found increased SIRPα expression in SAA-induced mice (Fig. 3A–D and Supplementry Fig. 6A–C). Concurrently, we observed significantly increased CD47 expression on live and apoptotic BM cells in SAA (Fig. 3E–G). Furthermore, IFNγ was critical for increased SIRPα–CD47 expression, as SAA-induced MIIG mice exhibited significantly reduced SIRPα^hi^ monocytes and CD47^+^ BM cells (Supplementry Fig. 6D–J). As MIIG mice are protected from disease, these findings support the role of aberrant CD47-SIRPα expression in disease progression.

CD47 is normally clustered in lipid rafts forming ‘punctates’ on the surface of live cells, allowing for high avidity binding to SIRPα (34). As cells undergo apoptosis, CD47 expression becomes diffuse, reducing the strength of CD47-SIRPα binding and allowing for efferocytosis of apoptotic cells (Fig. 3H). During SAA, an increase in CD47^+^ apoptotic cells coincided with a significant increase in CD47 MFI (Fig. 3I, J). Imaging flow cytometry of live and apoptotic BM cells revealed that apoptotic cells from RC mice exhibited diffuse CD47 staining, whereas apoptotic cells from SAA-induced mice retained a clustered phenotype (Fig. 3K). These data suggest that high avidity CD47-SIRPα engagement prevents the clearance of apoptotic cells during SAA.

### Decreased phagocytosis and efferocytosis in SAA

To examine whether phagocytes were functionally impaired during disease, SAA-induced mice were administered fluorescently(Dil)-labeled liposomes 9 dpst to evaluate phagocytotic capacity. SAA mice exhibited a decrease in Dil liposome-positive and Dil MFI on monocytes and macrophages by 10 dpst, suggesting impairments in phagocytosis (Supplementary Fig. 7).

To address whether efferocytosis was defective during SAA, we utilized phosphatidylserine (PS)-coated microparticles to mimic apoptotic cells. PS is a well-known “eat-me” signal recognized by tissue-resident phagocytes [39, 40]. BM from SAA-induced and RC mice was incubated with fluorescent PS-coated lipid microparticles (PS-MPs) and uptake determined via flow cytometry (Supplementary Fig. 8A, B). We observed a significant reduction in PS-MP^+^ monocytes and macrophages in marrow isolated from SAA-induced mice at 8 dpst (Supplementary Fig. 8C–E). Consistent with the role of impaired efferocytosis in disease progression, SAA-induced MIIG mice exhibited no change in efferocytosis compared to RC, whereas LC mice had significantly reduced uptake of PS-MPs (Supplementary Fig. 8F–G).

**Fig. 4:**
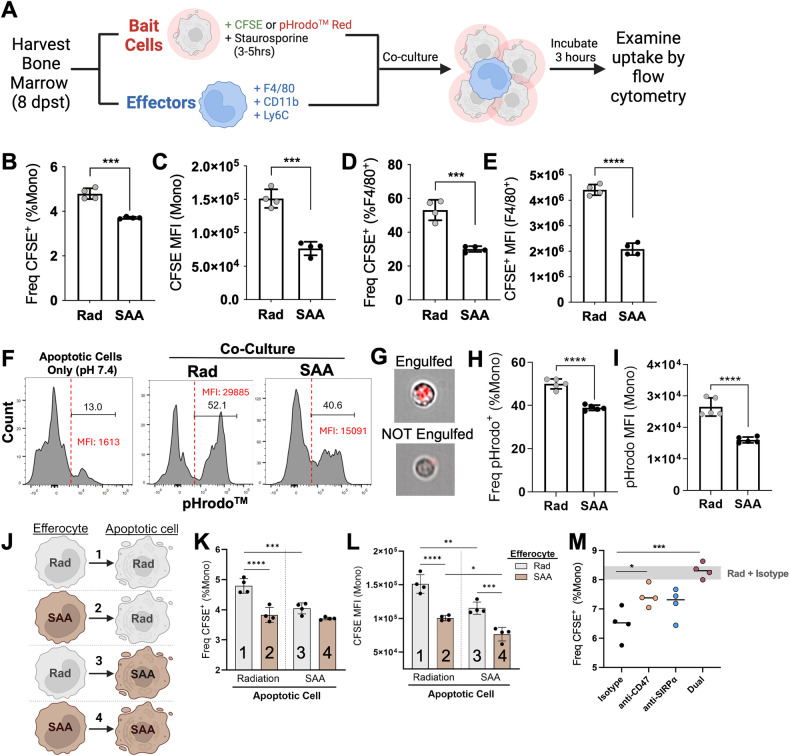
Enhanced SIRPα and CD47 expression is associated with impaired efferocytosis. **A** F1 mice were induced with SAA and BM harvested 8 dpst. BM cells were either stained with F4/80, CD11b, and Ly6C (effectors) or incubated with staurosporine to induce apoptosis and then stained with CFSE/pHrodo^TM^ Red (bait cells). The apoptotic “bait” cells were then fed to effector cells at a ratio of 4:1 (bait:effector) and incubated for 3 h before processing for flow cytometry. The frequency and MFI of CFSE^+^ bait cells in monocytes (**B**, **C**) and F4/80^+^ (**D**, **E**) effector cells. **F** Fluorescence emission of pHrodo in radiation and SAA monocytes. pHrodo-labeled apoptotic cells at neutral pH were used to make a cut-off point of fluorescence emission. **G** Representative cells from imaging flow cytometry of pHrodo fluorescence (red) after engulfment. The frequency (**H**) and MFI (**I**) of pHrodo in monocytes. Effector and bait cells from radiation control or SAA-induced mice were mixed-and-matched in culture, as shown in (**J**). The frequency (**K**) and MFI (**L**) of CFSE^+^ apoptotic cell uptake. The number on the bars represents the corresponding mix-and-match combination shown in (**J**). **M** Frequency of efferocytosis following incubation with 5 μg/mL anti-CD47 and/or 10 μg/mL anti-SIRPα. Data representative of one experiment showing mean ± SD, *n* = 4–5 mice per group, significance was determined using a Student’s t-test for **B**–**I**, and One/Two-way ANOVA with Tukey’s multiple comparison test for (**K**–**M**). *p < 0.05, ***p < 0.001, ****p < 0.0001.

To further examine efferocytosis, CFSE-labeled apoptotic “bait” cells from RC or SAA-induced mice were fed to “effector” BM cells (Fig. 4A). Monocytes and macrophages exhibited a significant decrease in the frequency and MFI of CFSE^+^ apoptotic cell uptake (Fig. 4B–E), indicative of impaired efferocytosis. To explore the engulfment and internalization, we utilized pHrodo^TM^ Red, a pH-sensitive fluorescent dye. After engulfment, pHrodo’s fluorescent intensity increases due to acidic conditions within the phagosome. pHrodo-labeled apoptotic cells at neutral pH were used to establish a threshold for identifying internalized cells (Fig. 4F, G). Monocytes from SAA-induced mice exhibited a significant reduction in the frequency and MFI of pHrodo, suggesting decreased engulfment of apoptotic cells (Fig. 4F–I).

Given increased SIRPα expression on monocytes and lack of CD47 dispersion on apoptotic cells, we next investigated how each cell subtype contributes to impaired efferocytosis. We mixed and matched labeled “bait” and “effector” cells from RC or SAA-induced mice (Fig. 4J). As expected, the group containing monocytes and apoptotic cells from RC exhibited the greatest efferocytic capacity (Fig. 4K–L, bar 1). All subsequent iterations containing a SAA-derived monocyte and/or bait cell demonstrated an equivalent reduction in efferocytosis (Fig. 4K, bar 2–4). However, the group containing both SAA subtypes exhibited a significant reduction in CFSE MFI, indicating a reduction in efferocytosis effeciency (Fig. 4L). These data support the hypothesis that an overexpression of SIRPα–CD47 contributes to impaired efferocytosis. In fact, neutralization of SIRPα–CD47 greatly enhanced apoptotic cell uptake and dual neutralization was able to fully recover efferocytosis by monocytes from SAA-induced mice (Fig. 4M). Together, these data support the role of aberrant SIRPα−CD47 in the progression of SAA.

### SIRPα-CD47 axis contributes to disease progression

To test whether the SIRPα−CD47 axis could be targeted therapeutically, SAA-induced mice were treated with anti-SIRPα neutralizing antibody 7, 9, 11, and 13 dpst (Supplementary Fig. 9A). We observed an increase in BM cellularity and a significant decrease in the frequency of dead cells and CD47^+^ apoptotic cells with anti-SIRPα treatment by 10 dpst (Supplementary Fig. 9B–E), supporting the notion that SIRPα limits efficient efferocytosis. However, as expected, anti-SIRPα treatment also exacerbated anemia during SAA by 14 dpst (Supplementary Fig. 9F-I), consistent with the role of CD47 as an important “marker of self” on healthy cells, especially red blood cells [41]. Therefore, while these data support the idea that the SIRPα−CD47 axis contributes to the accumulation of dead cells during SAA, the negative impact of anti-SIRPα on red blood cell circulation limits its therapeutic efficacy.

### Imbalance of lipid mediators that regulate inflammation resolution in SAA

**Fig. 5:**
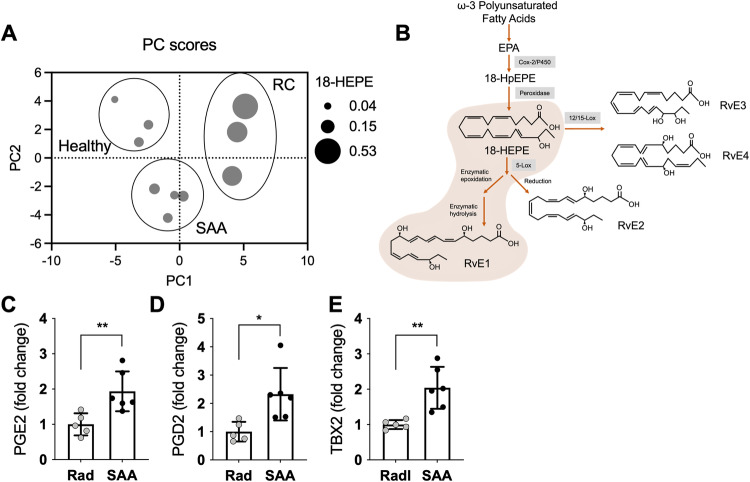
Imbalanced pro-Inflammatory- pro-resolving lipid mediators. Bone marrow was collected 3 or 8 dpst and analyzed by LC–MS/MS. **A** Principal component analysis of samples taken from mice without exposure to radiation (healthy), radiation only (RC), or radiation plus splenocytes to induce SAA (SAA) day 3 post radiation. Each dot represents data from a single mouse and the size of the dot reflects concentration of 18-HEPE. **B** Schematic of the biosynthesis of resolvins from ω-3 polyunsaturated fatty acids. Fold change in concentrations of PGE2 (**C**), PGD2 (**D**), or TXB2 (**E**) in the bone marrow of SAA mice, relative to RC, 8 dpst. Data representative of two independent experiments that included 2–3 mice each. Significance was determined using a Student’s *t*-test. *p < 0.05, **p < 0.01.

Since efferocytosis is an essential component of the resolution of inflammation, we next questioned whether disease progression correlated with differences in lipid mediators associated with resolution programs. We took an unbiased approach to evaluate the polyunsaturated fatty acid metabolome in BM of healthy, RC, and SAA-induced mice using LC-MS/MS. Principal component analysis (PCA) revealed that most of the variation (PC1; 43%) at 3 dpst was due to differences in mono-hydroxy eicosapentaenoic acids, including decreased 18-HEPE in SAA mice (Fig. 5A, Supplementry Fig. 10A). Limited 18-HEPE, the precursor to the E-series resolvins, may contribute to impaired generation of these SPMs in the marrow during SAA (Fig. 5B). By day 8, SAA-induced mice had significantly elevated prostaglandins (PGE2 and PGD2) and thromboxane B2 (TXB2) relative to RC (Fig. 5C–E, Supplementry Fig. 10B, supplementary Table 2). Therefore, omega-3-derived lipid precursors were decreased in the marrow during initiation of SAA, and disease progression correlated with unregulated prostanoid and thromboxane synthesis, supporting the conclusion that inflammation resolution kinetics are defective in SAA.

### Resolvin E1 provides therapeutic benefit in SAA

To address the therapeutic efficacy of SPMs for SAA, we first examined expression of key SPM receptors. We determined by flow cytometry that ChemR23 (*Cmklr1*), the receptor for E-series resolvins [42], was significantly upregulated on BM monocytes, neutrophils, and T cells, but not on macrophages or LK cells during SAA (Supplementry Fig. 11A–E). Gene expression analysis also demonstrated increased *Cmklr1* during SAA, especially on the Mono1 subset, which exhibited the greatest *Sirpa* expression (Supplementry Fig. 11F).

**Fig. 6:**
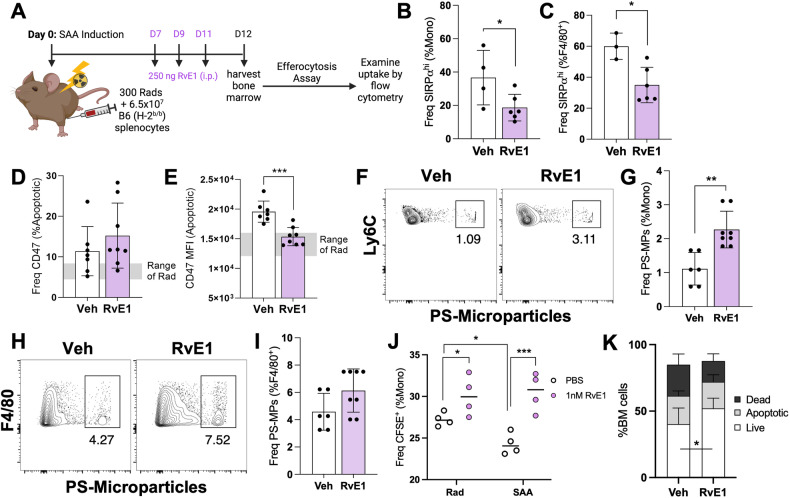
Exogenous RvE1 improves aberrant monocyte phenotype and efferocytosis. **A** Mice were induced to develop SAA and treated with 250 ng RvE1 days 7, 9, and 11 post induction. BM was harvested 12 dpst. The frequency of SIRPα on BM monocytes (**B**) and F4/80+ macrophages (**C**). Data representative of one experiment showing mean ± SD, *n* = 4–6 mice per group. Frequency (**D**) and MFI (**E**) of CD47 on apoptotic cells. Staining (**F**, **H**) and frequency (**G**, **I**) of PS-MPs on monocytes and F4/80^+^ cells. BM was harvested 8 dpst from RC and SAA-induced mice. BM cells were then labeled with CFSE, treated with staurosporine, and the apoptotic cells were then fed to effector BM cells with or without 1 nM RvE1. **J** The frequency of CFSE+ monocytes after overnight incubation. Data representative of one experiment with mean ± SD, *n* = 4 per group. **K** Frequency of live, apoptotic, and dead cells. Data from **A** to **I**, **K** represent two independent experiments with mean ± SD, *n* = 6–8 per group. Significance for **A**–**I** was determined using a Student’s t-test, and **J**, **K** via Two-way ANOVA with Tukey’s multiple comparison test. *p < 0.05, **p < 0.01, ***p < 0.001.

We first examined whether exogenous RvE1 could enhance efferocytosis during SAA by administering RvE1 or vehicle every other day starting 7 dpst, when the marrow is already hypocellular (Fig. 6A). RvE1 treatment decreased SIRPα expression on BM monocytes and macrophages. Although the frequency of CD47^+^ apoptotic cells was not changed, RvE1 treatment significantly reduced CD47 MFI, effectively restoring it to levels comparable to the RC (Fig. 6D, E). Decreased SIRPα-CD47 correlated with a significant increase in PS-MP^+^ monocytes and moderate increase in PS-MP^+^ F4/80^+^ cells at 12 dpst (Fig. 6F–I). To further validate RvE1’s impact on efferocytosis, CFSE^+^ apoptotic bait cells were fed to effector BM cells with or without 1 nM RvE1 (Fig. 6J). Consistent with the PS-MPs, RvE1 significantly increased the uptake of CFSE^+^ apoptotic cells by monocytes, which correlated with an improvement in the frequency of live BM cells (Fig. 6K). Together, these data support the conclusion that RvE1 improves efferocytosis in SAA-induced mice.

**Fig. 7:**
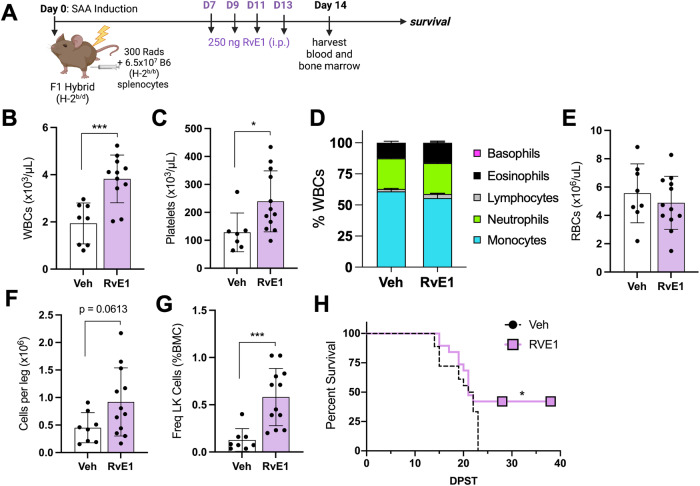
Exogenous RvE1 improves platelets, BM cellularity and survival. **A** SAA-induced F1 hybrid mice were treated with 250 ng of RvE1 days 7, 9, 11, and 13 post induction. BM and blood was evaluated 14 dpst. The frequency of all WBCs (**B**), platelets (**C**), WBC breakdown (**D**), and total RBCs (**E**) are shown. Total BM cellularity (**F**) and frequency of LK cells (**G**) in F1 mice treated with vehicle or RvE1. Data representative of two independent experiments showing mean ± SD, *n* = 8–12 per group. Significance was determined using a Student’s *t*-test. *p < 0.05, ***p < 0.001. **H** Percent survival 28 or 37 days post induction; SAA mice treated with vehicle (black dashed line) *n* = *18*; SAA mice treated with RvE1 (purple line) *n* = *19*. Data were pooled from three independent experiments.

**Fig. 8:**
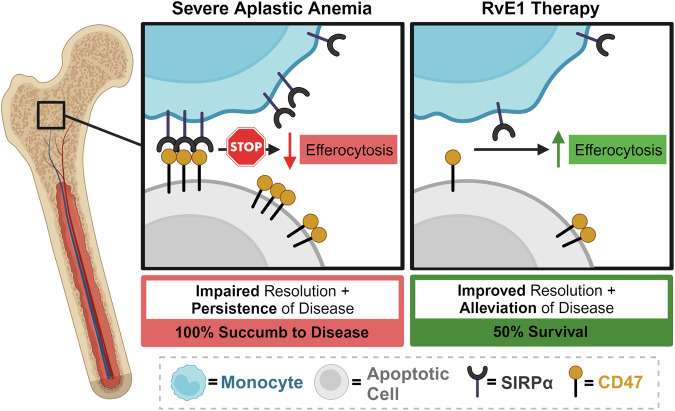
RvE1 therapy reduces SIRPα-CD47, increases efferocytosis, and improves survival. Schematic model illustrating the role of impaired efferocytosis in dysfunctional inflammation resolution and the development of SAA. An increase in SIRPα^hi^ monocytes and CD47^+^ apoptotic cells prevent clearance of apoptotic and dead cells, which is associated with disease progression (left panel). Exogenous RvE1 treatment decreases SIRPα^hi^ monocytes and CD47+ apoptotic cells, correlating with increased efferocytosis and improved survival (right panel).

RvE1 treatment also improved WBC count and platelets by 14 dpst (Fig. 7A–C). While RvE1 did not improve lymphopenia, anemia, and only mildly limited SAA-induced monocytosis (Fig. 7D, E), BM cellularity, LK cells, and HSC progenitor populations were increased (Fig. 7F, G; Supplementary Fig. 12). Importantly, RvE1 significantly improved survival when mice were administered therapeutic doses of RvE1 (Fig. 7H). Together, our data demonstrate that RvE1 therapy decreases SIRPα-CD47 and improves efferocytic function, which correlated with protection against SAA-induced mortality (Fig. 8). These findings suggest that dysfunctional inflammation resolution is a novel therapeutic target for improving treatments for SAA patients.

## Discussion

Current treatments for SAA rely on HSC transplantation and IST, though IST has a high refractory rate. Recently approved Etrombopag, a thrombopoietin receptor agonist, improved response times in combination with IST, but did not show overall improvement in older patients or those with severe disease [43]. Preclinical studies using the Jak1/2 inhibitor Ruxolitinib demonstrated effectiveness in a murine model of SAA with reduced T lymphocytes in the marrow [44], however toxicity associated with Ruxolitinib has been noted [45, 46]. While suppressing inflammation is an important target for treating SAA, we reasoned that understanding mechanisms underlying non-resolving inflammation may reveal novel targets for treatment.

Efferocytosis promotes inflammation resolution by removing apoptotic cells, releasing anti-inflammatory mediators, and driving SPM biosynthesis [47, 48]. Impairments in resolution underly several chronic inflammatory diseases [49–52], however, these pathways have not been investigated in BMF. We found an accumulation of CD47^+^ apoptotic cells that correlated with expansion of SIRPα^hi^ monocytes, suggesting deficiencies in cell clearance.

SPMs actively drive the resolution of inflammation and restore homeostasis [19]. Class switching of lipid mediators, from pro-inflammatory to pro-resolving, is essential for resolution to occur [53]. We have demonstrated that SAA-induced mice exhibit an imbalance of pro-inflammatory to pro-resolving mediators with exuberant prostanoid synthesis. SPM biosynthesis occurs via transcellular mechanisms requiring close contact of cells expressing distinct enzymes. During SAA the BM becomes profoundly hypocellular, potentially preventing SPM generation. Reduced SPM biosynthesis or signaling has been linked with increased inflammation in various disease settings [54–56]. A lack of SPM production and increased prostanoids, combined with dysregulated unalamation [57], appear to contribute to persistent inflammation observed during SAA. Indeed, a prostanoid storm limited efferocytosis in vitro and in atherosclerotic plaques [58], supporting the notion that sustained elevated prostanoids, without a compensatory increase in SPMs, may contribute to defective efferocytosis in the BM during SAA.

Our finding that the E-series resolvins precursor was reduced while ChemR23, the receptor for RvE1, was increased on monocytes and neutrophils in SAA, provided strong rationale for targeting inflammation resolution during BMF. Indeed, RvE1 treatment improved efferocytosis, platelet count, BM cellularity, and survival even when treatment was initiated when the BM is already hypocellular. Therapies aimed at improving resolution, perhaps in conjunction with lower dosing of IST, may provide a more effective and safe treatment for SAA. Moreover, improving resolution may contribute to more durable responses to IST that improve long-term outcomes.

### Supplementary information

Supplementary Material

## Data Availability

Methods for flow cytometric and lipidomic analysis are described in detail in the Supplementry materials. Single-cell RNA sequencing data is available at GEO under accession number GSE237388. For original data please contact the authors.
