# Supplementary material for: Resolvin E1 improves efferocytosis and rescues severe aplastic anemia in mice
Source: Cell Death Dis. 2024 May 9;15(5):324. doi: 10.1038/s41419-024-06705-7 (PMC11082201; doi:10.1038/s41419-024-06705-7)
Supplement: Supplementary file 1 — Supplementary Material [file 41419_2024_6705_MOESM1_ESM.pptx]

## Slide 1
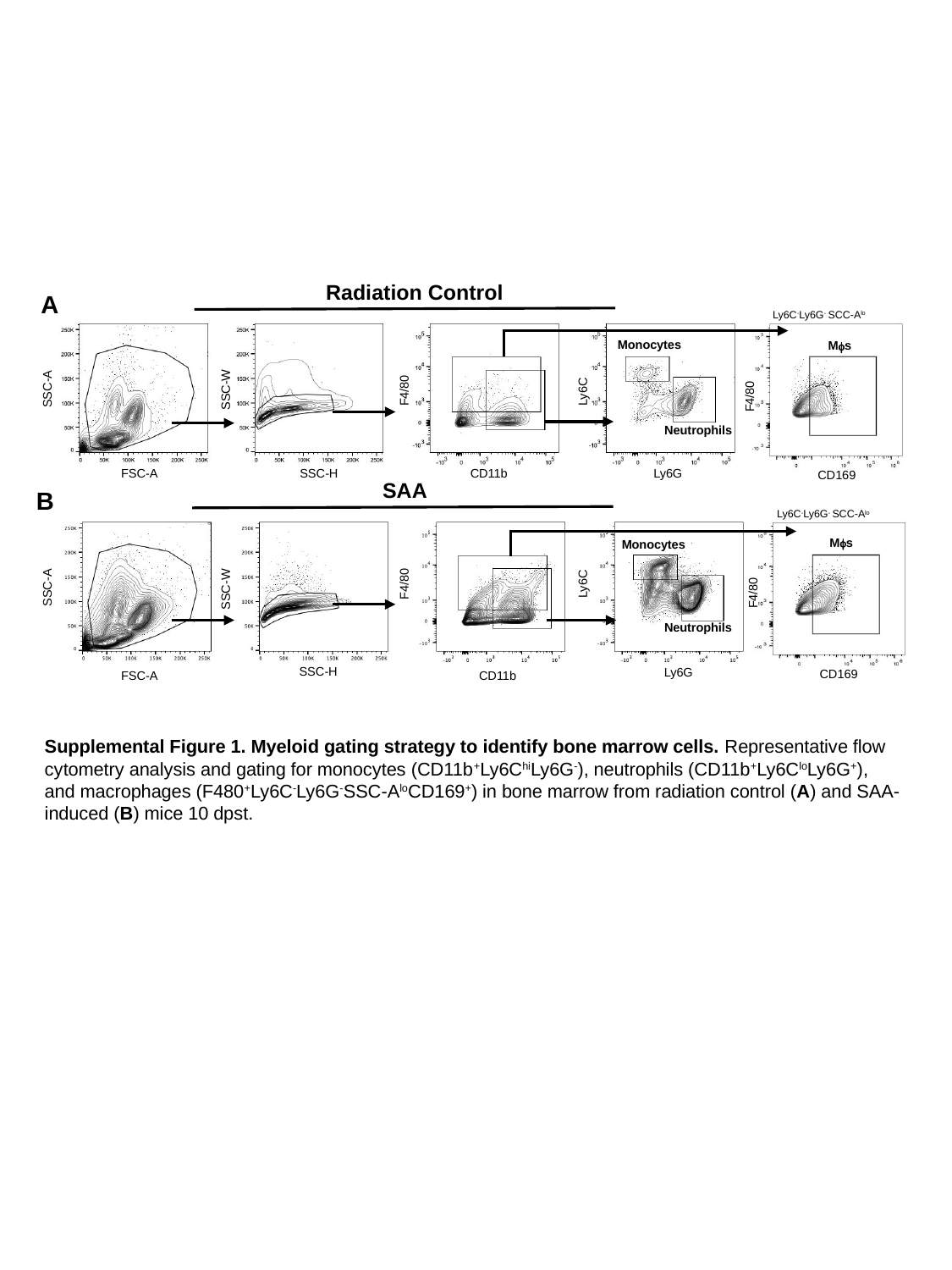

Radiation Control
A
Ly6C-Ly6G- SCC-Alo
Monocytes
Mfs
SSC-A
SSC-W
F4/80
Ly6C
F4/80
Neutrophils
SSC-H
CD11b
Ly6G
FSC-A
CD169
SAA
B
Ly6C-Ly6G- SCC-Alo
Mfs
Monocytes
Ly6C
F4/80
SSC-A
SSC-W
F4/80
Neutrophils
SSC-H
Ly6G
CD169
CD11b
FSC-A
Supplemental Figure 1. Myeloid gating strategy to identify bone marrow cells. Representative flow cytometry analysis and gating for monocytes (CD11b+Ly6ChiLy6G-), neutrophils (CD11b+Ly6CloLy6G+), and macrophages (F480+Ly6C-Ly6G-SSC-AloCD169+) in bone marrow from radiation control (A) and SAA-induced (B) mice 10 dpst.

## Slide 2
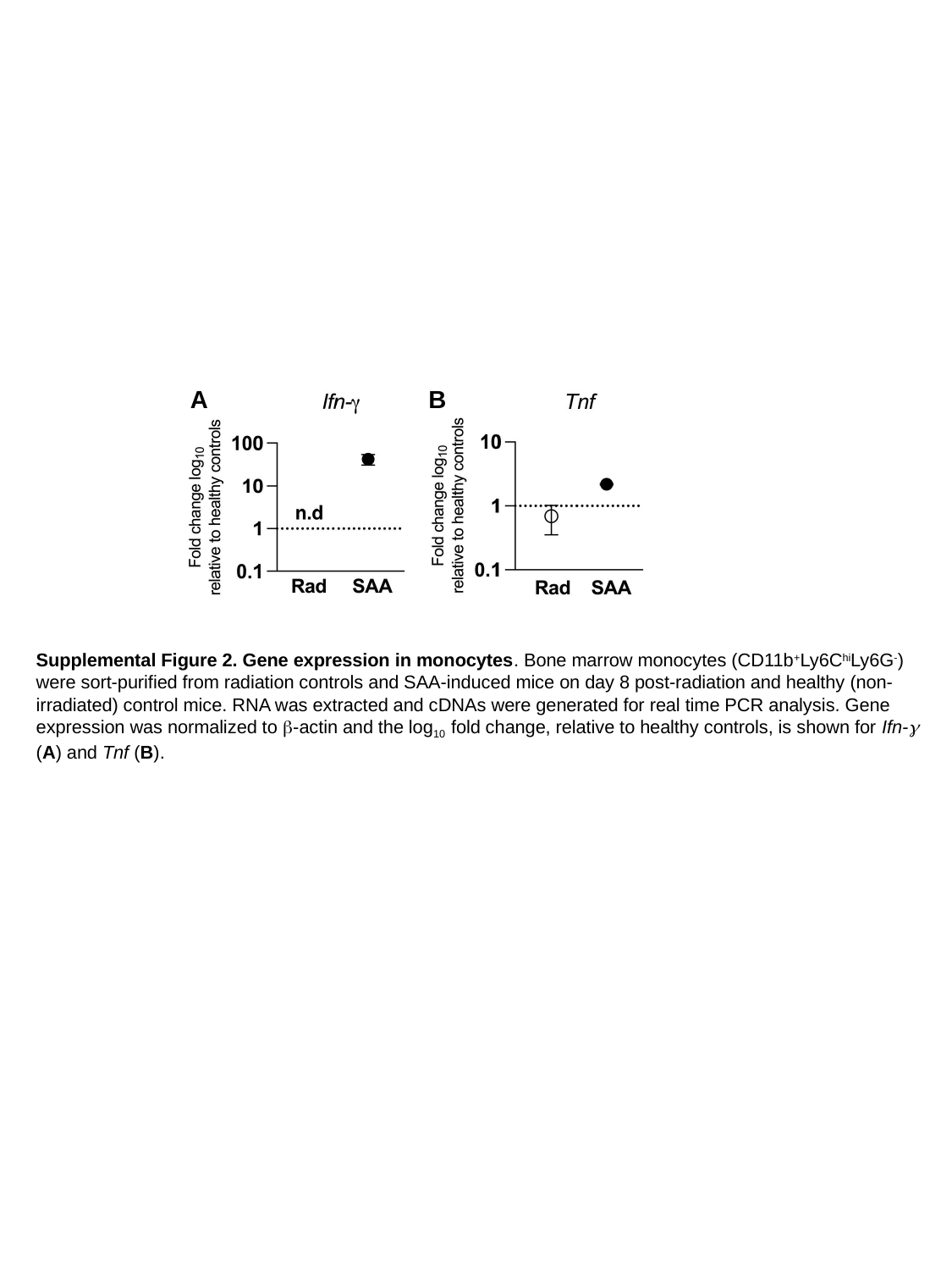

A
B
Supplemental Figure 2. Gene expression in monocytes. Bone marrow monocytes (CD11b+Ly6ChiLy6G-) were sort-purified from radiation controls and SAA-induced mice on day 8 post-radiation and healthy (non-irradiated) control mice. RNA was extracted and cDNAs were generated for real time PCR analysis. Gene expression was normalized to b-actin and the log10 fold change, relative to healthy controls, is shown for Ifn-g (A) and Tnf (B).

## Slide 3
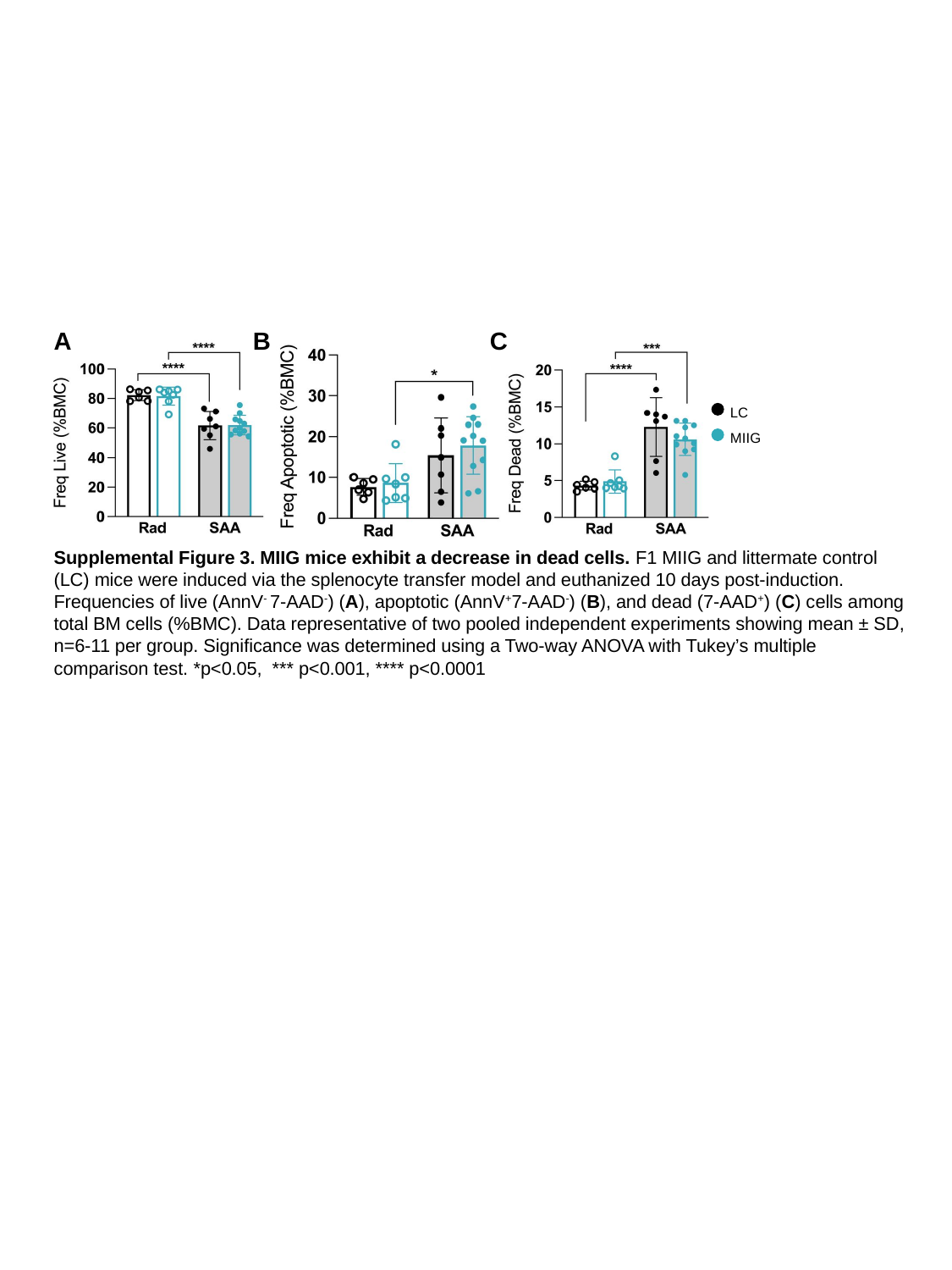

A
B
C
LC
MIIG
Supplemental Figure 3. MIIG mice exhibit a decrease in dead cells. F1 MIIG and littermate control (LC) mice were induced via the splenocyte transfer model and euthanized 10 days post-induction. Frequencies of live (AnnV- 7-AAD-) (A), apoptotic (AnnV+7-AAD-) (B), and dead (7-AAD+) (C) cells among total BM cells (%BMC). Data representative of two pooled independent experiments showing mean ± SD, n=6-11 per group. Significance was determined using a Two-way ANOVA with Tukey’s multiple comparison test. *p<0.05, *** p<0.001, **** p<0.0001

## Slide 4
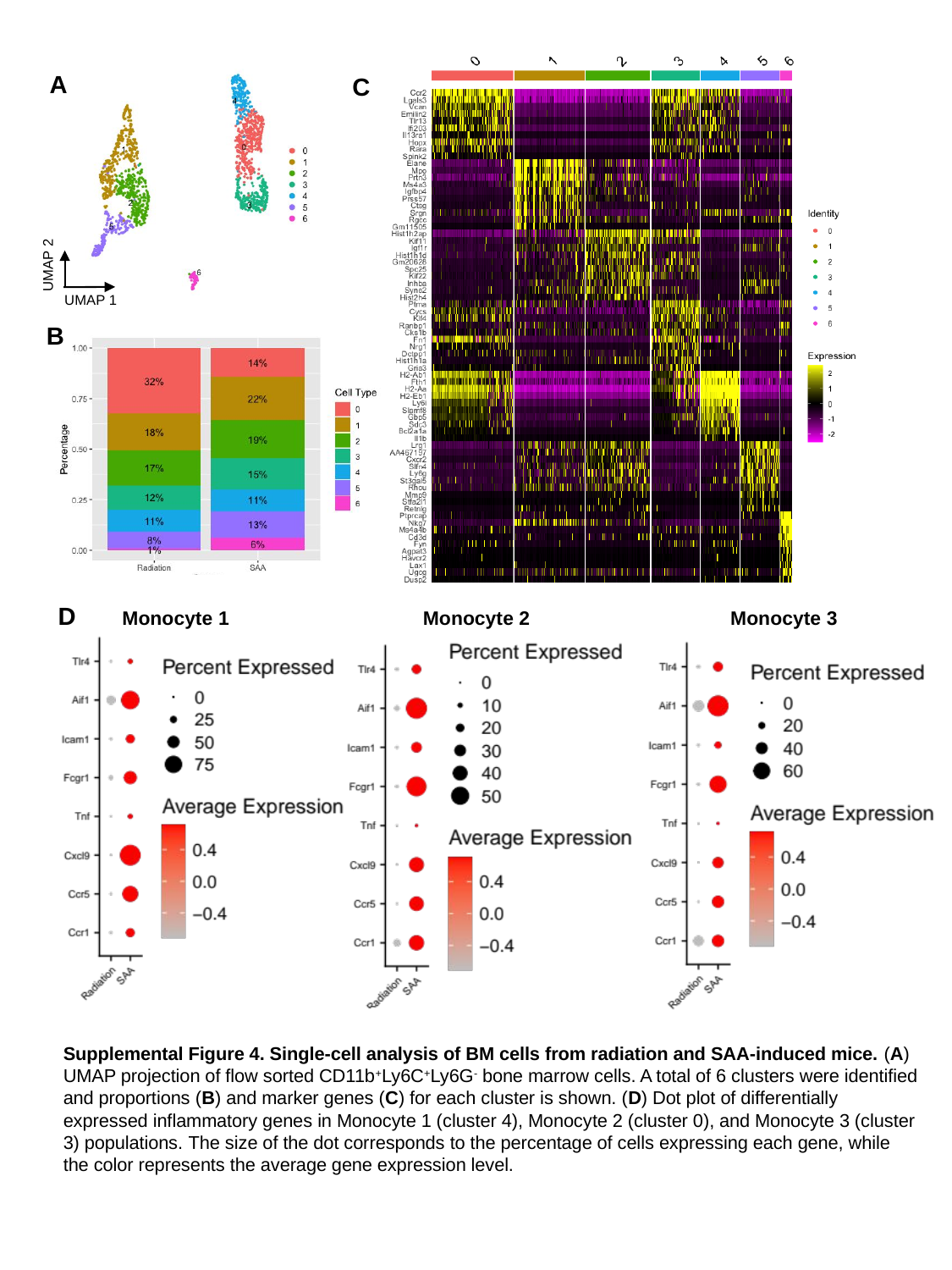

A
C
UMAP 2
UMAP 1
B
D
Monocyte 1
Monocyte 2
Monocyte 3
Supplemental Figure 4. Single-cell analysis of BM cells from radiation and SAA-induced mice. (A) UMAP projection of flow sorted CD11b+Ly6C+Ly6G- bone marrow cells. A total of 6 clusters were identified and proportions (B) and marker genes (C) for each cluster is shown. (D) Dot plot of differentially expressed inflammatory genes in Monocyte 1 (cluster 4), Monocyte 2 (cluster 0), and Monocyte 3 (cluster 3) populations. The size of the dot corresponds to the percentage of cells expressing each gene, while the color represents the average gene expression level.

## Slide 5
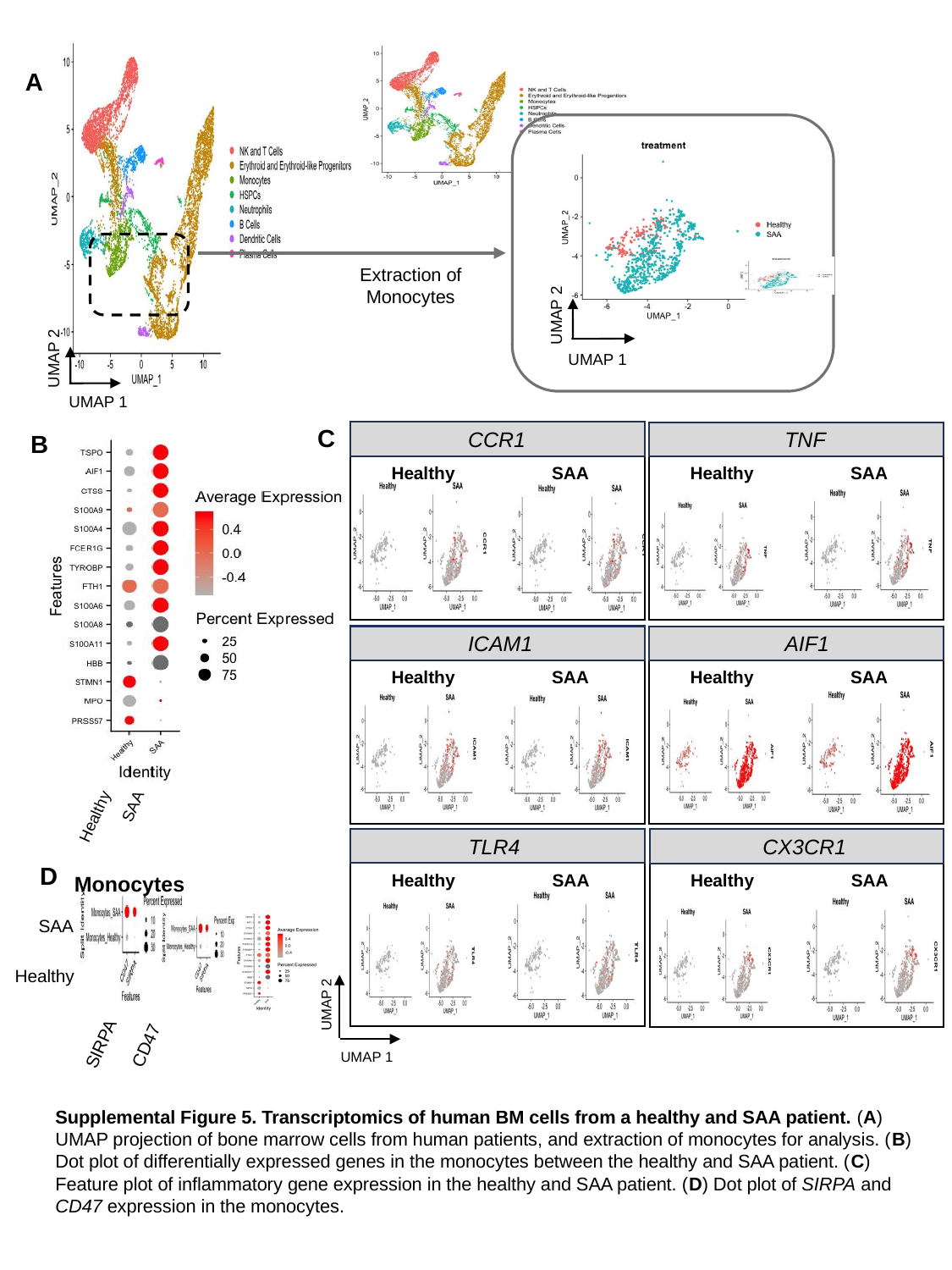

A
Extraction of Monocytes
UMAP 2
UMAP 1
UMAP 2
UMAP 1
C
CCR1
TNF
B
SAA
Healthy
SAA
Healthy
ICAM1
AIF1
SAA
Healthy
SAA
Healthy
SAA
Healthy
TLR4
CX3CR1
D
SAA
Healthy
SAA
Healthy
Monocytes
SAA
Healthy
UMAP 2
SIRPA
CD47
UMAP 1
Supplemental Figure 5. Transcriptomics of human BM cells from a healthy and SAA patient. (A) UMAP projection of bone marrow cells from human patients, and extraction of monocytes for analysis. (B) Dot plot of differentially expressed genes in the monocytes between the healthy and SAA patient. (C) Feature plot of inflammatory gene expression in the healthy and SAA patient. (D) Dot plot of SIRPA and CD47 expression in the monocytes.

## Slide 6
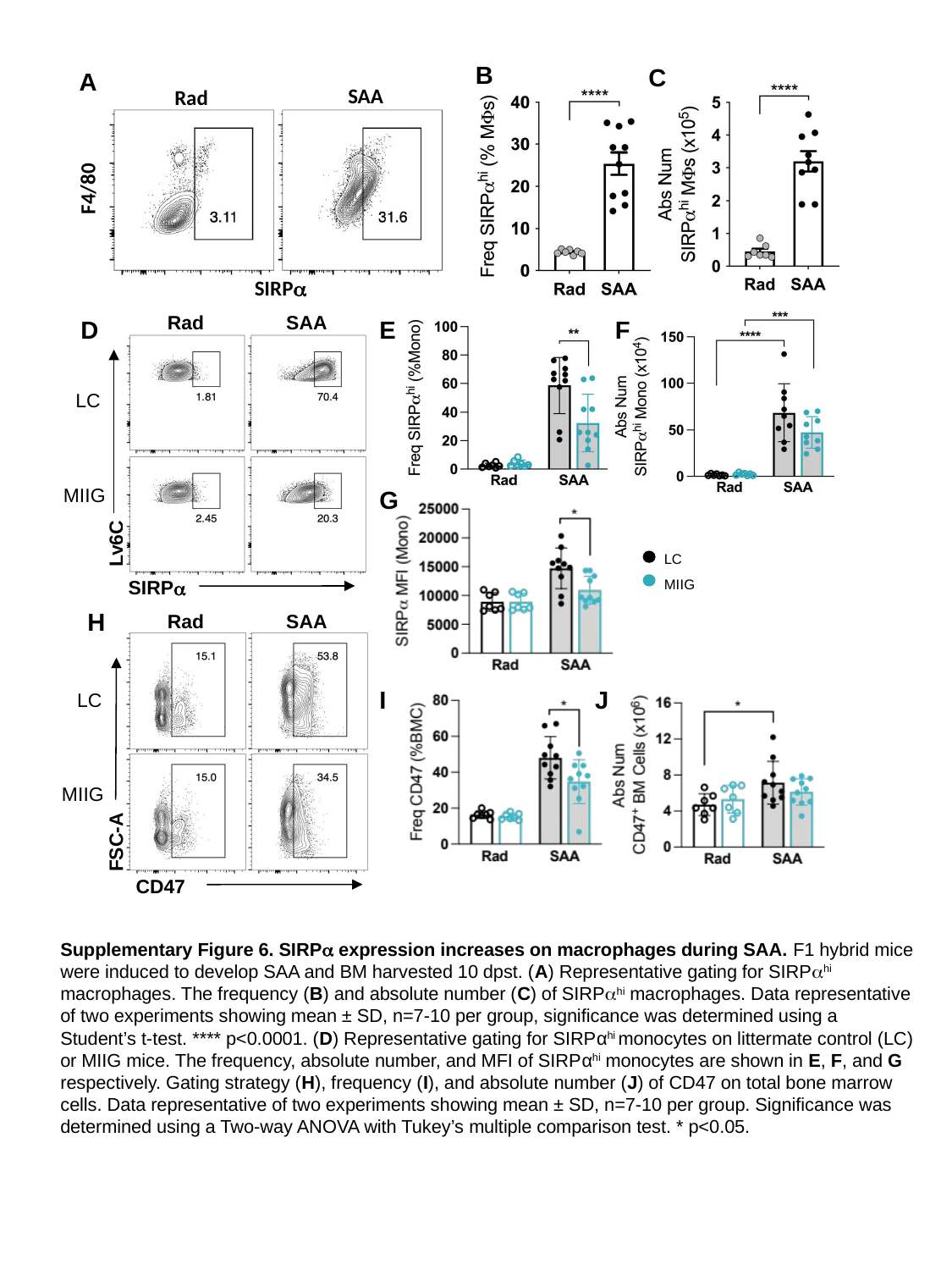

B
C
A
SAA
Rad
F4/80
SIRPa
Rad SAA
E
D
F
LC
MIIG
G
Ly6C
LC
MIIG
SIRPa
H
Rad SAA
I
J
LC
MIIG
FSC-A
CD47
Supplementary Figure 6. SIRPa expression increases on macrophages during SAA. F1 hybrid mice were induced to develop SAA and BM harvested 10 dpst. (A) Representative gating for SIRPahi macrophages. The frequency (B) and absolute number (C) of SIRPahi macrophages. Data representative of two experiments showing mean ± SD, n=7-10 per group, significance was determined using a Student’s t-test. **** p<0.0001. (D) Representative gating for SIRPαhi monocytes on littermate control (LC) or MIIG mice. The frequency, absolute number, and MFI of SIRPαhi monocytes are shown in E, F, and G respectively. Gating strategy (H), frequency (I), and absolute number (J) of CD47 on total bone marrow cells. Data representative of two experiments showing mean ± SD, n=7-10 per group. Significance was determined using a Two-way ANOVA with Tukey’s multiple comparison test. * p<0.05.

## Slide 7
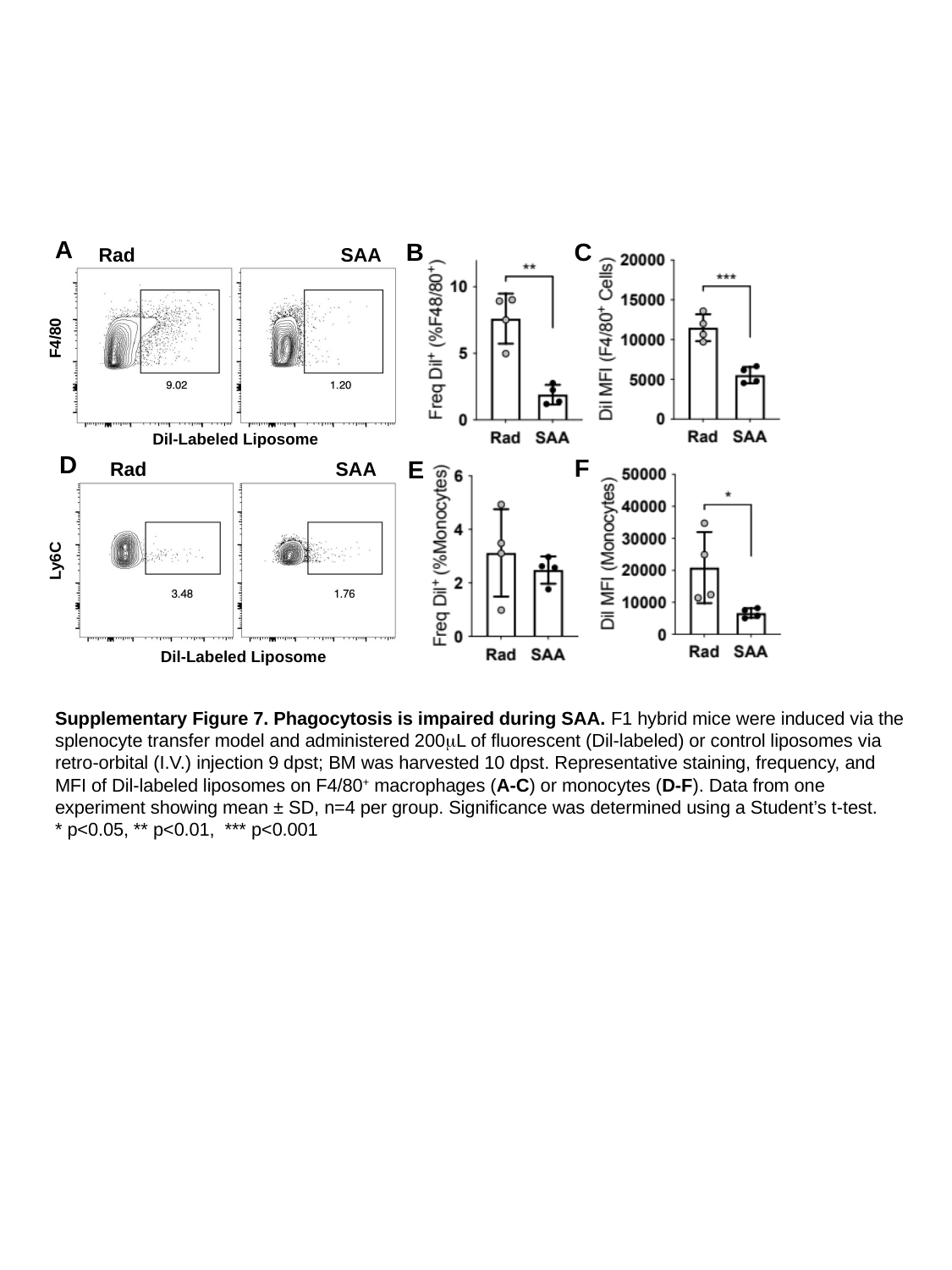

A
C
B
Rad	 SAA
F4/80
Dil-Labeled Liposome
D
F
E
Rad	 SAA
Ly6C
Dil-Labeled Liposome
Supplementary Figure 7. Phagocytosis is impaired during SAA. F1 hybrid mice were induced via the splenocyte transfer model and administered 200mL of fluorescent (Dil-labeled) or control liposomes via retro-orbital (I.V.) injection 9 dpst; BM was harvested 10 dpst. Representative staining, frequency, and MFI of Dil-labeled liposomes on F4/80+ macrophages (A-C) or monocytes (D-F). Data from one experiment showing mean ± SD, n=4 per group. Significance was determined using a Student’s t-test.
* p<0.05, ** p<0.01, *** p<0.001

## Slide 8
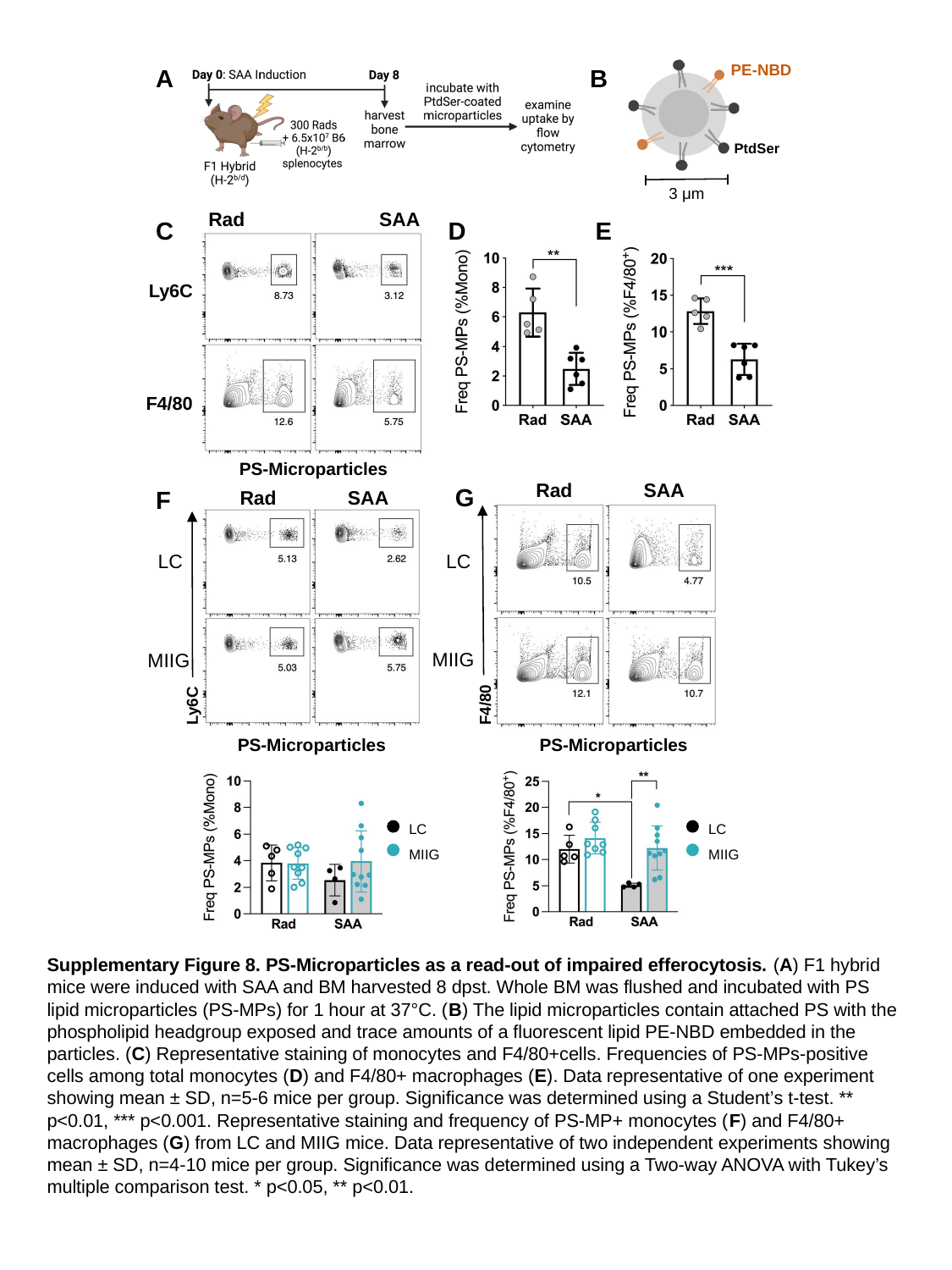

PE-NBD
PtdSer
3 μm
A
B
Rad 	 SAA
C
D
E
Ly6C
F4/80
PS-Microparticles
Rad SAA
G
F
Rad SAA
LC
LC
MIIG
MIIG
F4/80
Ly6C
PS-Microparticles
PS-Microparticles
LC
MIIG
LC
MIIG
Supplementary Figure 8. PS-Microparticles as a read-out of impaired efferocytosis. (A) F1 hybrid mice were induced with SAA and BM harvested 8 dpst. Whole BM was flushed and incubated with PS lipid microparticles (PS-MPs) for 1 hour at 37°C. (B) The lipid microparticles contain attached PS with the phospholipid headgroup exposed and trace amounts of a fluorescent lipid PE-NBD embedded in the particles. (C) Representative staining of monocytes and F4/80+cells. Frequencies of PS-MPs-positive cells among total monocytes (D) and F4/80+ macrophages (E). Data representative of one experiment showing mean ± SD, n=5-6 mice per group. Significance was determined using a Student’s t-test. ** p<0.01, *** p<0.001. Representative staining and frequency of PS-MP+ monocytes (F) and F4/80+ macrophages (G) from LC and MIIG mice. Data representative of two independent experiments showing mean ± SD, n=4-10 mice per group. Significance was determined using a Two-way ANOVA with Tukey’s multiple comparison test. * p<0.05, ** p<0.01.

## Slide 9
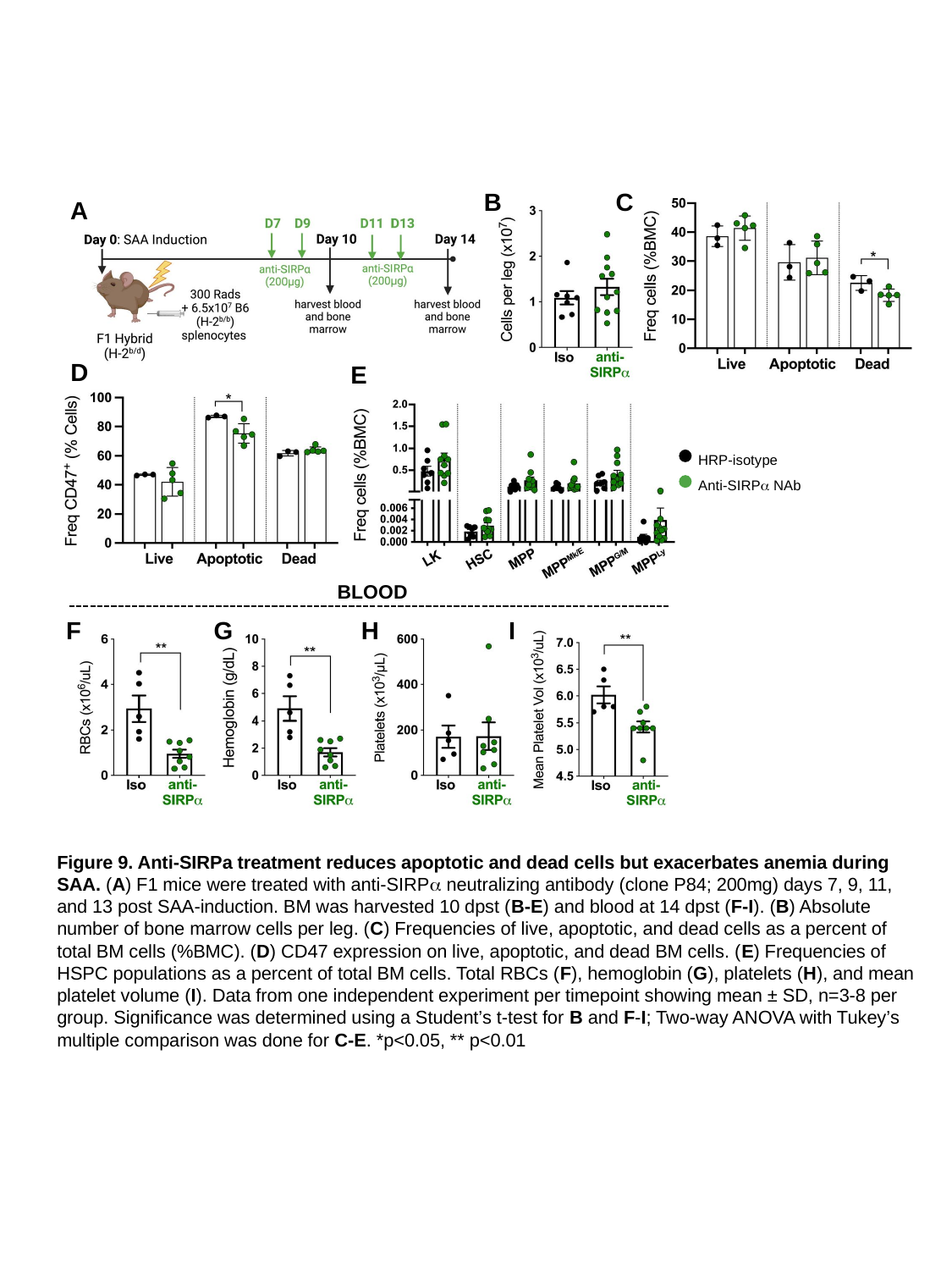

C
B
A
D
E
HRP-isotype
Anti-SIRPa NAb
BLOOD
F
G
H
I
Figure 9. Anti-SIRPa treatment reduces apoptotic and dead cells but exacerbates anemia during SAA. (A) F1 mice were treated with anti-SIRPa neutralizing antibody (clone P84; 200mg) days 7, 9, 11, and 13 post SAA-induction. BM was harvested 10 dpst (B-E) and blood at 14 dpst (F-I). (B) Absolute number of bone marrow cells per leg. (C) Frequencies of live, apoptotic, and dead cells as a percent of total BM cells (%BMC). (D) CD47 expression on live, apoptotic, and dead BM cells. (E) Frequencies of HSPC populations as a percent of total BM cells. Total RBCs (F), hemoglobin (G), platelets (H), and mean platelet volume (I). Data from one independent experiment per timepoint showing mean ± SD, n=3-8 per group. Significance was determined using a Student’s t-test for B and F-I; Two-way ANOVA with Tukey’s multiple comparison was done for C-E. *p<0.05, ** p<0.01

## Slide 10
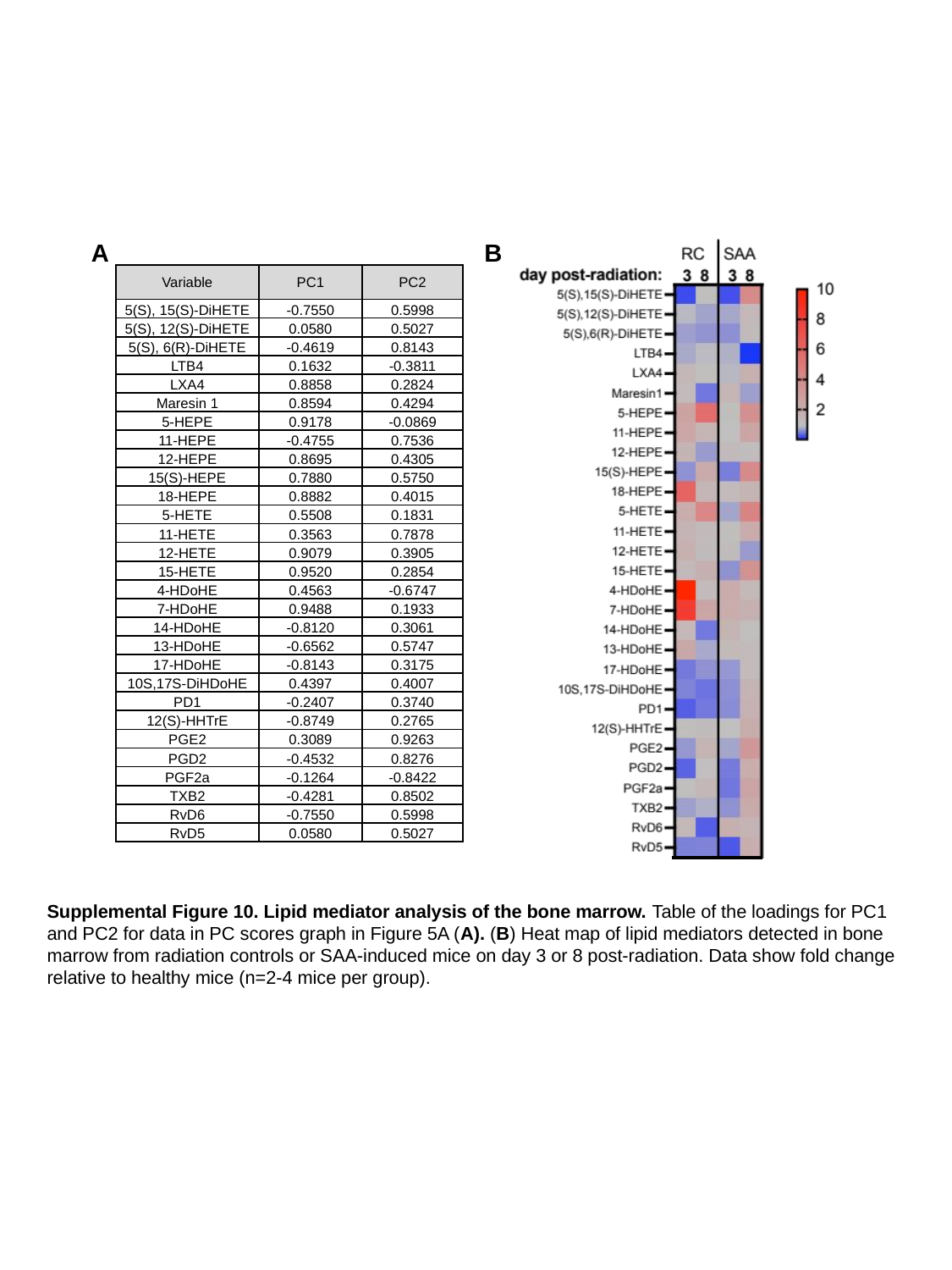

A
B
| Variable | PC1 | PC2 |
| --- | --- | --- |
| 5(S), 15(S)-DiHETE | -0.7550 | 0.5998 |
| 5(S), 12(S)-DiHETE | 0.0580 | 0.5027 |
| 5(S), 6(R)-DiHETE | -0.4619 | 0.8143 |
| LTB4 | 0.1632 | -0.3811 |
| LXA4 | 0.8858 | 0.2824 |
| Maresin 1 | 0.8594 | 0.4294 |
| 5-HEPE | 0.9178 | -0.0869 |
| 11-HEPE | -0.4755 | 0.7536 |
| 12-HEPE | 0.8695 | 0.4305 |
| 15(S)-HEPE | 0.7880 | 0.5750 |
| 18-HEPE | 0.8882 | 0.4015 |
| 5-HETE | 0.5508 | 0.1831 |
| 11-HETE | 0.3563 | 0.7878 |
| 12-HETE | 0.9079 | 0.3905 |
| 15-HETE | 0.9520 | 0.2854 |
| 4-HDoHE | 0.4563 | -0.6747 |
| 7-HDoHE | 0.9488 | 0.1933 |
| 14-HDoHE | -0.8120 | 0.3061 |
| 13-HDoHE | -0.6562 | 0.5747 |
| 17-HDoHE | -0.8143 | 0.3175 |
| 10S,17S-DiHDoHE | 0.4397 | 0.4007 |
| PD1 | -0.2407 | 0.3740 |
| 12(S)-HHTrE | -0.8749 | 0.2765 |
| PGE2 | 0.3089 | 0.9263 |
| PGD2 | -0.4532 | 0.8276 |
| PGF2a | -0.1264 | -0.8422 |
| TXB2 | -0.4281 | 0.8502 |
| RvD6 | -0.7550 | 0.5998 |
| RvD5 | 0.0580 | 0.5027 |
Supplemental Figure 10. Lipid mediator analysis of the bone marrow. Table of the loadings for PC1 and PC2 for data in PC scores graph in Figure 5A (A). (B) Heat map of lipid mediators detected in bone marrow from radiation controls or SAA-induced mice on day 3 or 8 post-radiation. Data show fold change relative to healthy mice (n=2-4 mice per group).

## Slide 11
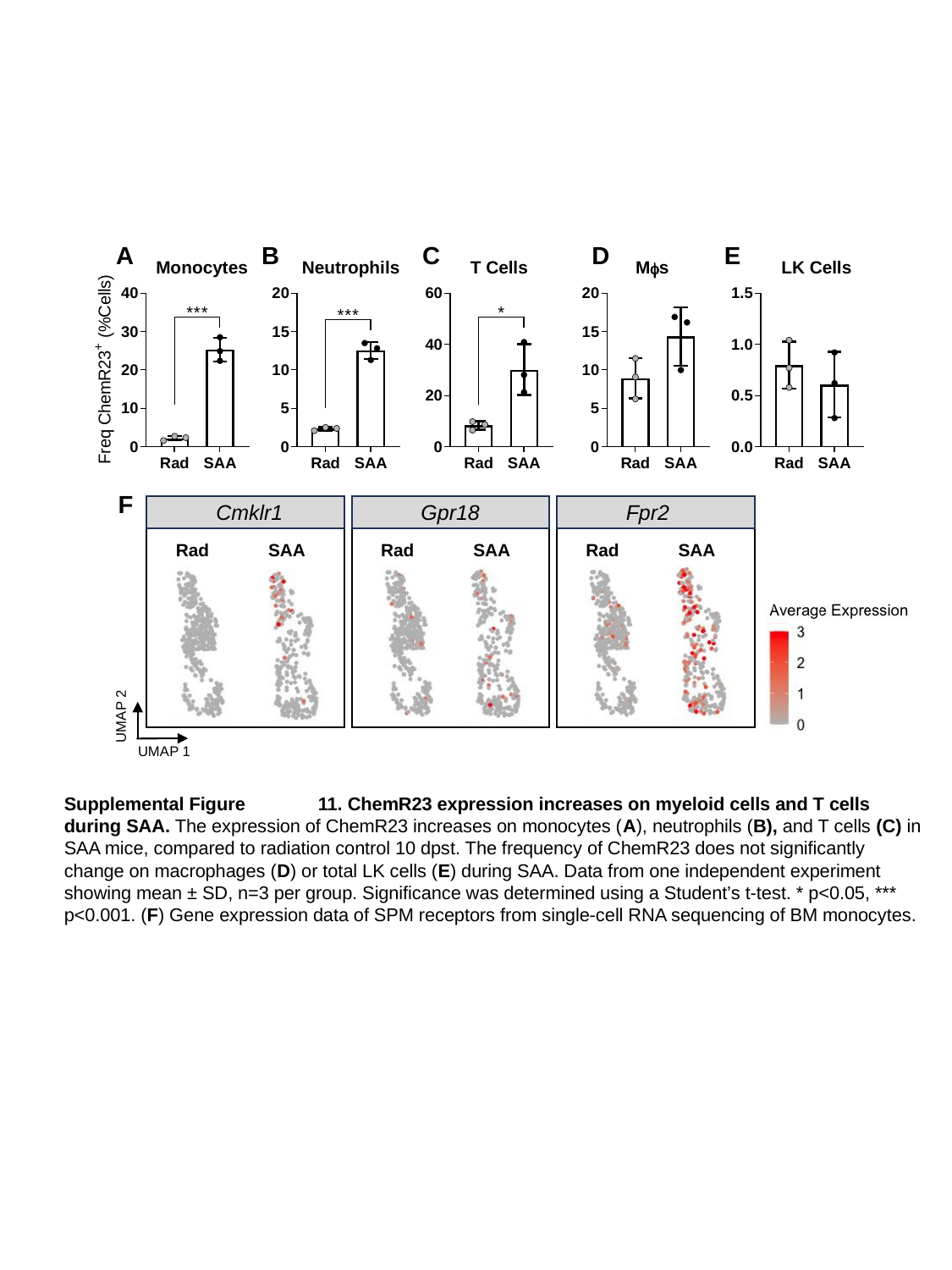

A
B
C
D
E
Monocytes
Neutrophils
T Cells
Mfs
LK Cells
F
Cmklr1
Gpr18
Fpr2
Rad
SAA
Rad
SAA
Rad
SAA
UMAP 2
UMAP 1
Supplemental Figure 	11. ChemR23 expression increases on myeloid cells and T cells during SAA. The expression of ChemR23 increases on monocytes (A), neutrophils (B), and T cells (C) in SAA mice, compared to radiation control 10 dpst. The frequency of ChemR23 does not significantly change on macrophages (D) or total LK cells (E) during SAA. Data from one independent experiment showing mean ± SD, n=3 per group. Significance was determined using a Student’s t-test. * p<0.05, *** p<0.001. (F) Gene expression data of SPM receptors from single-cell RNA sequencing of BM monocytes.

## Slide 12
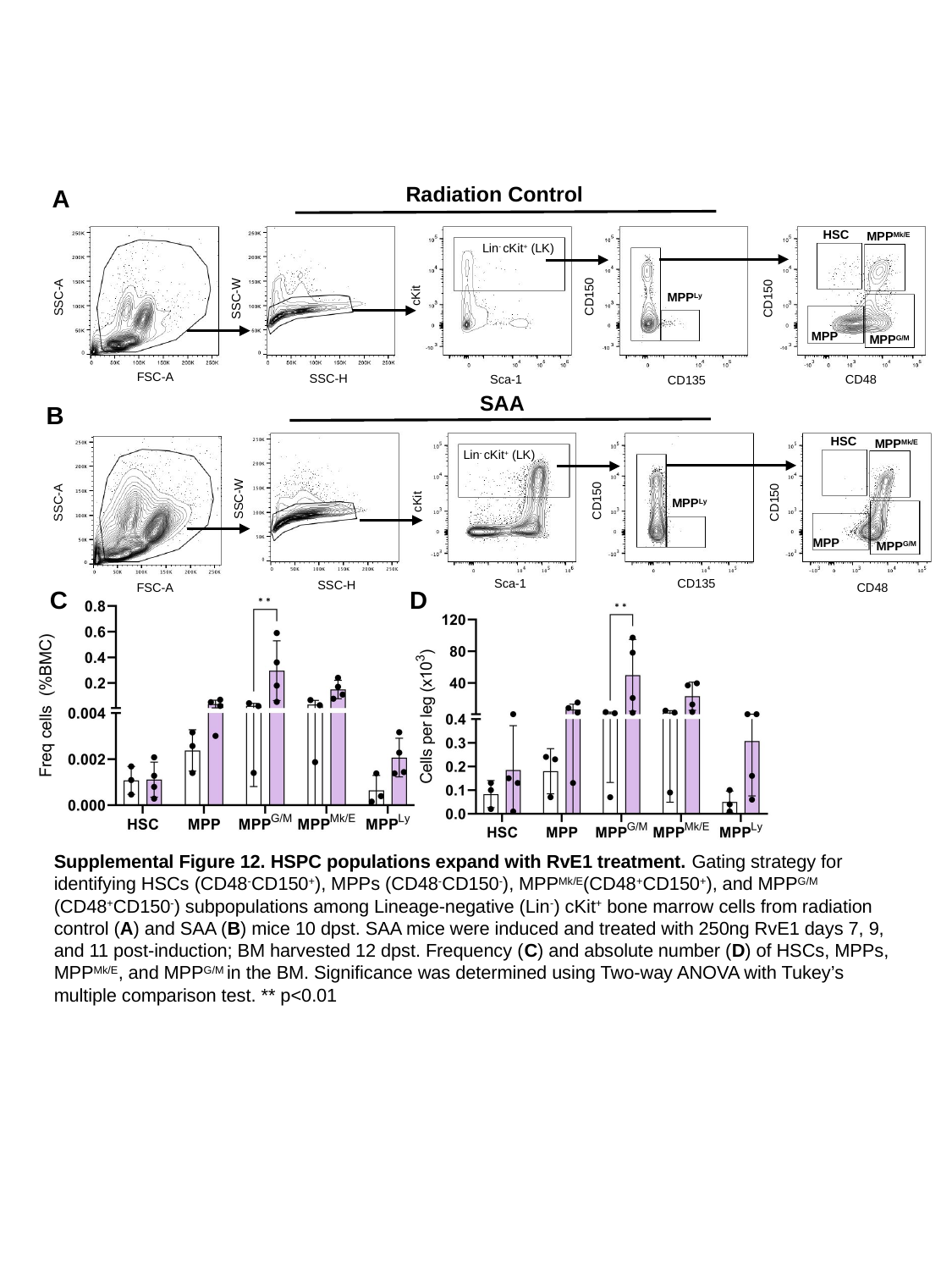

Radiation Control
A
HSC
MPPMk/E
Lin- cKit+ (LK)
cKit
CD150
CD150
MPPLy
SSC-A
SSC-W
MPP
MPPG/M
FSC-A
SSC-H
Sca-1
CD48
CD135
SAA
B
HSC
MPPMk/E
Lin- cKit+ (LK)
cKit
SSC-W
CD150
CD150
SSC-A
MPPLy
MPP
MPPG/M
CD135
Sca-1
SSC-H
FSC-A
CD48
D
C
Supplemental Figure 12. HSPC populations expand with RvE1 treatment. Gating strategy for identifying HSCs (CD48-CD150+), MPPs (CD48-CD150-), MPPMk/E(CD48+CD150+), and MPPG/M (CD48+CD150-) subpopulations among Lineage-negative (Lin-) cKit+ bone marrow cells from radiation control (A) and SAA (B) mice 10 dpst. SAA mice were induced and treated with 250ng RvE1 days 7, 9, and 11 post-induction; BM harvested 12 dpst. Frequency (C) and absolute number (D) of HSCs, MPPs, MPPMk/E, and MPPG/M in the BM. Significance was determined using Two-way ANOVA with Tukey’s multiple comparison test. ** p<0.01

## Slide 13
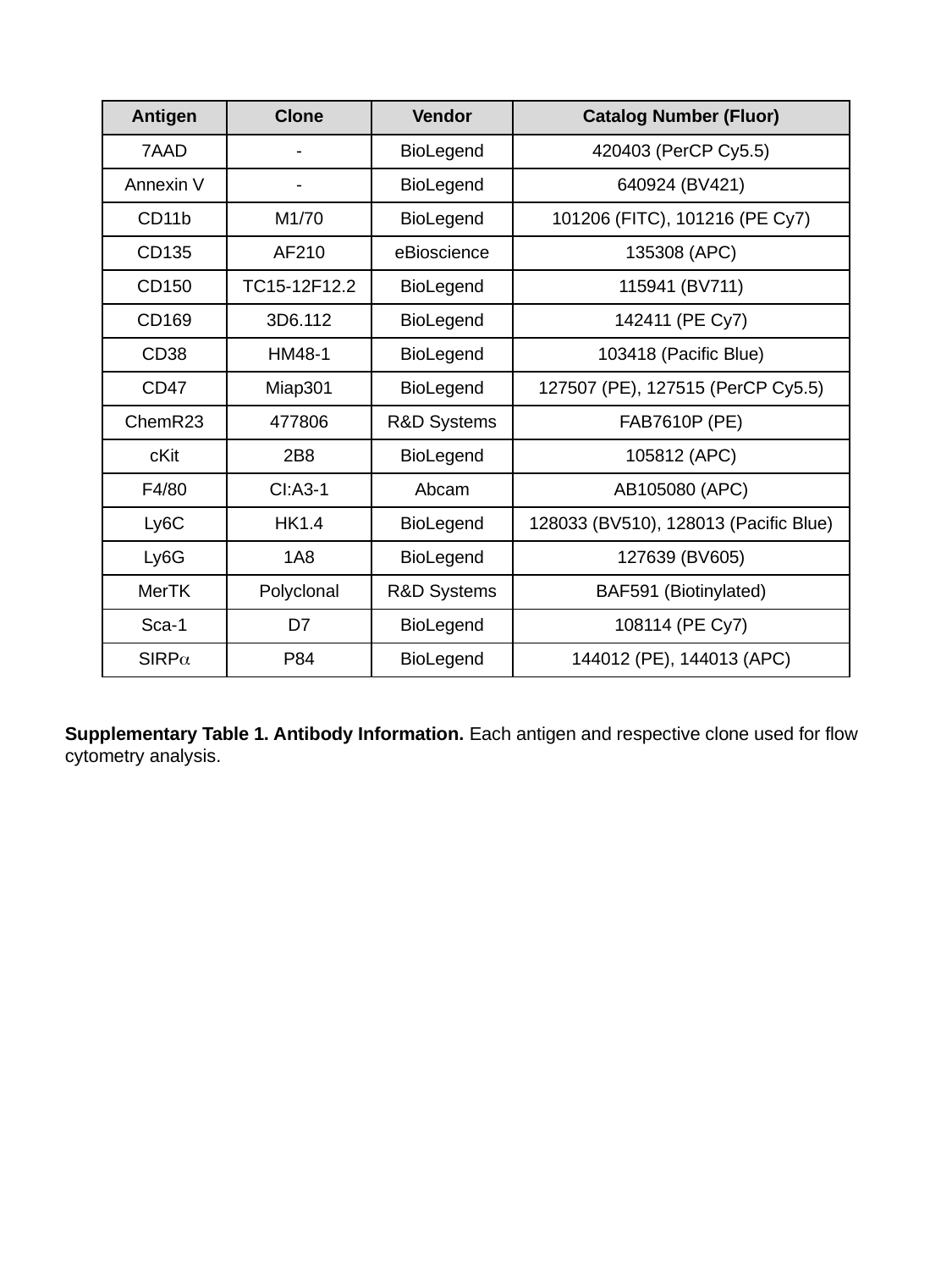

| Antigen | Clone | Vendor | Catalog Number (Fluor) |
| --- | --- | --- | --- |
| 7AAD | - | BioLegend | 420403 (PerCP Cy5.5) |
| Annexin V | - | BioLegend | 640924 (BV421) |
| CD11b | M1/70 | BioLegend | 101206 (FITC), 101216 (PE Cy7) |
| CD135 | AF210 | eBioscience | 135308 (APC) |
| CD150 | TC15-12F12.2 | BioLegend | 115941 (BV711) |
| CD169 | 3D6.112 | BioLegend | 142411 (PE Cy7) |
| CD38 | HM48-1 | BioLegend | 103418 (Pacific Blue) |
| CD47 | Miap301 | BioLegend | 127507 (PE), 127515 (PerCP Cy5.5) |
| ChemR23 | 477806 | R&D Systems | FAB7610P (PE) |
| cKit | 2B8 | BioLegend | 105812 (APC) |
| F4/80 | CI:A3-1 | Abcam | AB105080 (APC) |
| Ly6C | HK1.4 | BioLegend | 128033 (BV510), 128013 (Pacific Blue) |
| Ly6G | 1A8 | BioLegend | 127639 (BV605) |
| MerTK | Polyclonal | R&D Systems | BAF591 (Biotinylated) |
| Sca-1 | D7 | BioLegend | 108114 (PE Cy7) |
| SIRPa | P84 | BioLegend | 144012 (PE), 144013 (APC) |
Supplementary Table 1. Antibody Information. Each antigen and respective clone used for flow cytometry analysis.

## Slide 14
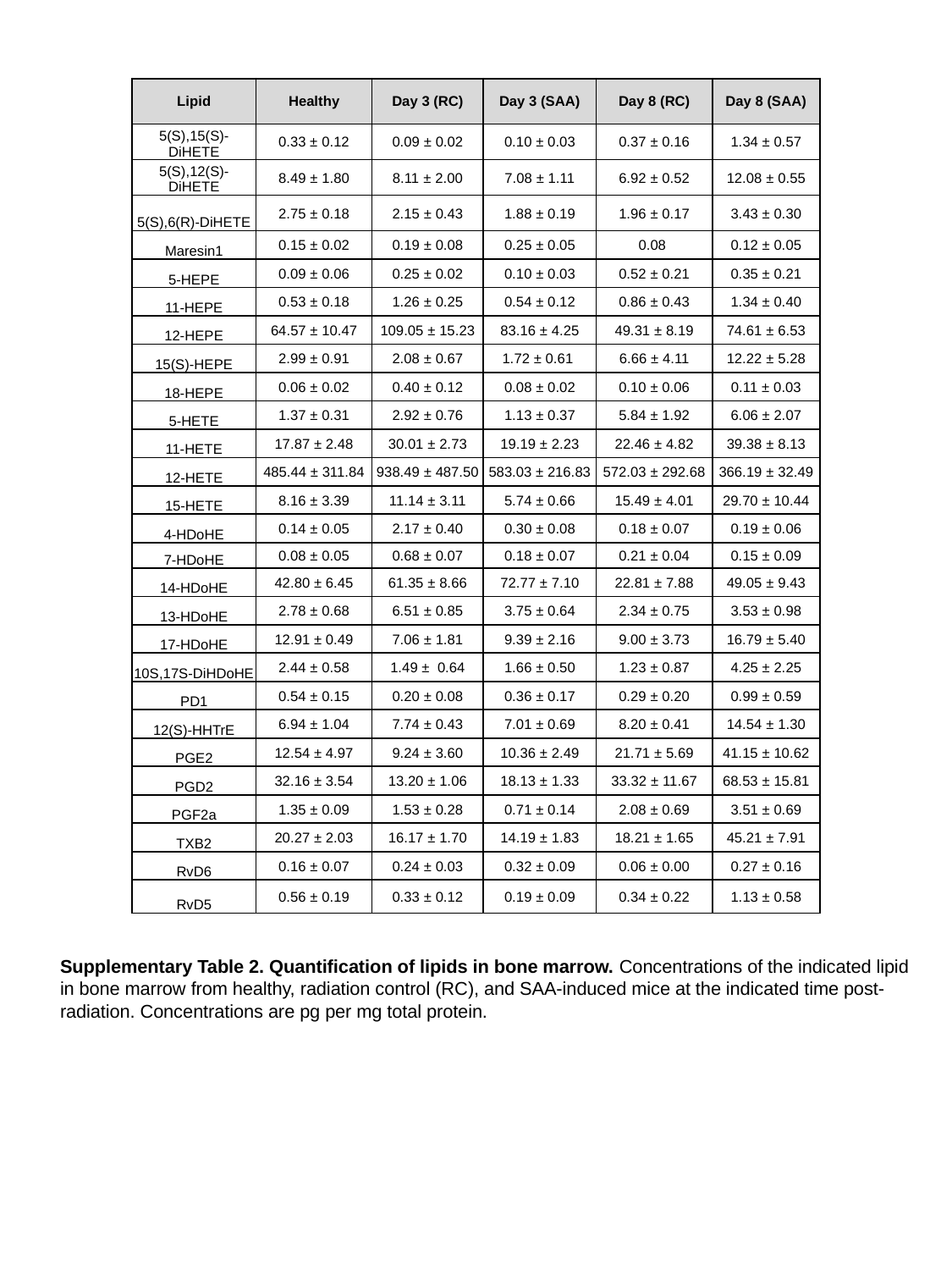

| Lipid | Healthy | Day 3 (RC) | Day 3 (SAA) | Day 8 (RC) | Day 8 (SAA) |
| --- | --- | --- | --- | --- | --- |
| 5(S),15(S)-DiHETE | 0.33 ± 0.12 | 0.09 ± 0.02 | 0.10 ± 0.03 | 0.37 ± 0.16 | 1.34 ± 0.57 |
| 5(S),12(S)-DiHETE | 8.49 ± 1.80 | 8.11 ± 2.00 | 7.08 ± 1.11 | 6.92 ± 0.52 | 12.08 ± 0.55 |
| 5(S),6(R)-DiHETE | 2.75 ± 0.18 | 2.15 ± 0.43 | 1.88 ± 0.19 | 1.96 ± 0.17 | 3.43 ± 0.30 |
| Maresin1 | 0.15 ± 0.02 | 0.19 ± 0.08 | 0.25 ± 0.05 | 0.08 | 0.12 ± 0.05 |
| 5-HEPE | 0.09 ± 0.06 | 0.25 ± 0.02 | 0.10 ± 0.03 | 0.52 ± 0.21 | 0.35 ± 0.21 |
| 11-HEPE | 0.53 ± 0.18 | 1.26 ± 0.25 | 0.54 ± 0.12 | 0.86 ± 0.43 | 1.34 ± 0.40 |
| 12-HEPE | 64.57 ± 10.47 | 109.05 ± 15.23 | 83.16 ± 4.25 | 49.31 ± 8.19 | 74.61 ± 6.53 |
| 15(S)-HEPE | 2.99 ± 0.91 | 2.08 ± 0.67 | 1.72 ± 0.61 | 6.66 ± 4.11 | 12.22 ± 5.28 |
| 18-HEPE | 0.06 ± 0.02 | 0.40 ± 0.12 | 0.08 ± 0.02 | 0.10 ± 0.06 | 0.11 ± 0.03 |
| 5-HETE | 1.37 ± 0.31 | 2.92 ± 0.76 | 1.13 ± 0.37 | 5.84 ± 1.92 | 6.06 ± 2.07 |
| 11-HETE | 17.87 ± 2.48 | 30.01 ± 2.73 | 19.19 ± 2.23 | 22.46 ± 4.82 | 39.38 ± 8.13 |
| 12-HETE | 485.44 ± 311.84 | 938.49 ± 487.50 | 583.03 ± 216.83 | 572.03 ± 292.68 | 366.19 ± 32.49 |
| 15-HETE | 8.16 ± 3.39 | 11.14 ± 3.11 | 5.74 ± 0.66 | 15.49 ± 4.01 | 29.70 ± 10.44 |
| 4-HDoHE | 0.14 ± 0.05 | 2.17 ± 0.40 | 0.30 ± 0.08 | 0.18 ± 0.07 | 0.19 ± 0.06 |
| 7-HDoHE | 0.08 ± 0.05 | 0.68 ± 0.07 | 0.18 ± 0.07 | 0.21 ± 0.04 | 0.15 ± 0.09 |
| 14-HDoHE | 42.80 ± 6.45 | 61.35 ± 8.66 | 72.77 ± 7.10 | 22.81 ± 7.88 | 49.05 ± 9.43 |
| 13-HDoHE | 2.78 ± 0.68 | 6.51 ± 0.85 | 3.75 ± 0.64 | 2.34 ± 0.75 | 3.53 ± 0.98 |
| 17-HDoHE | 12.91 ± 0.49 | 7.06 ± 1.81 | 9.39 ± 2.16 | 9.00 ± 3.73 | 16.79 ± 5.40 |
| 10S,17S-DiHDoHE | 2.44 ± 0.58 | 1.49 ± 0.64 | 1.66 ± 0.50 | 1.23 ± 0.87 | 4.25 ± 2.25 |
| PD1 | 0.54 ± 0.15 | 0.20 ± 0.08 | 0.36 ± 0.17 | 0.29 ± 0.20 | 0.99 ± 0.59 |
| 12(S)-HHTrE | 6.94 ± 1.04 | 7.74 ± 0.43 | 7.01 ± 0.69 | 8.20 ± 0.41 | 14.54 ± 1.30 |
| PGE2 | 12.54 ± 4.97 | 9.24 ± 3.60 | 10.36 ± 2.49 | 21.71 ± 5.69 | 41.15 ± 10.62 |
| PGD2 | 32.16 ± 3.54 | 13.20 ± 1.06 | 18.13 ± 1.33 | 33.32 ± 11.67 | 68.53 ± 15.81 |
| PGF2a | 1.35 ± 0.09 | 1.53 ± 0.28 | 0.71 ± 0.14 | 2.08 ± 0.69 | 3.51 ± 0.69 |
| TXB2 | 20.27 ± 2.03 | 16.17 ± 1.70 | 14.19 ± 1.83 | 18.21 ± 1.65 | 45.21 ± 7.91 |
| RvD6 | 0.16 ± 0.07 | 0.24 ± 0.03 | 0.32 ± 0.09 | 0.06 ± 0.00 | 0.27 ± 0.16 |
| RvD5 | 0.56 ± 0.19 | 0.33 ± 0.12 | 0.19 ± 0.09 | 0.34 ± 0.22 | 1.13 ± 0.58 |
Supplementary Table 2. Quantification of lipids in bone marrow. Concentrations of the indicated lipid in bone marrow from healthy, radiation control (RC), and SAA-induced mice at the indicated time post-radiation. Concentrations are pg per mg total protein.
